# Advancements in Electrochromic Technology for Multifunctional Flexible Devices

**DOI:** 10.3390/ma18132964

**Published:** 2025-06-23

**Authors:** Alice Marciel, Joel Borges, Luiz Pereira, Rui F. Silva, Manuel Graça

**Affiliations:** 1 i3N and Department of Physics, University of Aveiro, Campus Universitário de Santiago, 3810-193 Aveiro, Portugalmpfg@ua.pt (M.G.); 2 CICECO, Aveiro Institute of Materials, Department of Materials and Ceramic Engineering, University of Aveiro, 3810-193 Aveiro, Portugal; 3 Physics Center of Minho and Porto Universities (CF-UM-UP), University of Minho, Campus de Azurém, 4800-058 Guimarães, Portugal

**Keywords:** electrochromism, electrochromic materials, flexibility, multifunctional electrochromic devices, energy-efficient systems

## Abstract

The design and investigation of electrochromic devices have advanced significantly, including distinct applications such as self-charged smart windows, aerospace interactive windows, low power flexible and ecofriendly displays, automatic dimming rearview, wearable smart textiles, military and civilian camouflage systems, electrochromic sensors, among others. Although significant progress has been made in related fields, achieving the full potential of electrochromic devices to meet the standards of maturity and practical applications remains a persistent challenge. Electrochromic devices are typically multilayered structures that can be designed as either rigid or flexible systems, depending on the type of substrate employed. Conventional electrochromic devices comprise layered structures that include transparent electrodes, electrochromic materials, ionic conductors, and ion storage materials. On the other hand, multifunctional systems integrate bifunctional materials or distinct functional layers to simultaneously achieve optical modulation and additional capabilities such as energy storage. The development of advanced materials, comprehensive electrochemical kinetic analysis, the optimization and advancement of process techniques and deposition methods, and innovative device designs are active areas of extensive global research. This review focuses on the recent advances in multifunctional electrochromic materials and devices with particular emphasis on the integration of electrochromic technology with other functional technologies. It further identifies current challenges, proposes potential solutions, and outlines future research directions focused on advancing this technology in both niche and scalable applications.

## 1. Introduction

Electrochromic technology has been pivotal in creating low-cost, energy-efficient, and environmentally friendly solutions that contribute to sustainable development. In particular, it provides a direct response to the growing demand for smarter energy use in various areas, from construction to consumer electronics. Electrochromism refers to the ability of certain materials to reversibly and persistently alter their optical properties under the application of an electric field [1,2,3]. Furthermore, electrochromic materials with the ability to store energy are highly desirable. These materials have demonstrated a wide range of promising applications, including, for instance, displays [4,5,6], anti-glare car rear view mirrors [7,8,9], smart windows [10,11,12,13], and new ones that are emerging, such as wearables [14,15,16,17], sensors [18,19,20,21,22,23,24], adaptive military and civil camouflage [25], and energy storage/conversion systems [26,27,28].

Conventional electrochromic devices have a multilayer structure (typically a five-layer structure) consisting of transparent electrodes, electrochromic layers, ionic conductors, and an ion storage layer. Rigid and flexible electrochromic devices (ECDs) have similar structures, differing mainly in substrate design and manufacturing complexity. Glass [4,6,29,30] or acrylic [31] are examples of rigid substrates, whereas polyethylene terephthalate (PET), such as in Refs. [13,32,33], polycarbonate (PC) [34], silicone polymer polydimethylsiloxane (PDMS) [35], thermoplastic polyurethane (TPU) [36], and nylon fibres [37] are examples of flexible ones. Substrates have a structural function, providing an adequate base to accommodate and support the subsequent layers. Depending on the type of electrochromic device, desirable substrate properties may include low weight, deformability, impact resistance, and environmental and chemical resistance, among others [31,38]. Transparent electrodes (working and counter electrodes, for electron and charge transfer) are placed on the top and bottom of the device in the case of transmissive devices and on the side that is to be viewed for reflecting devices [39]. Recognized candidates as electrodes include doped metal oxide nanostructures, which are the most common (among others), indium tin oxide (ITO) [32,40,41] and fluorine-doped tin dioxide (FTO) [42], aluminum doped zin oxide (AZO) [43,44], metal nanowires (silver nanowires (Ag NWs), copper nanowires (Cu NWs)) [45,46,47], graphene conductive layers [48,49,50], carbon nanotubes [51], metal grids [51,52], poly(3,4-ethylenedioxythiophene)-poly(styrenesulfonate) (PEDOT:PSS) [53,54], and composite materials such as AgNWs/rGO (reduced graphene oxide) [55], Ag (silver)-Au(gold) core–shell nanowire networks [56], and graphene silver nanowire composite [57]. The electrochromic layer composed of electrochromic materials is the core of an electrochromic device (ECD) and is responsible for the colour change and optical modulation. Electrochromic material can be inorganic (e.g., WO_3_ [58,59], V_2_O_5_ [60,61], MoO_x_ [32,62], NiO [63], Nb_2_O_5_ [40,64,65], Nb-Mo-O [41], Prussian blue [66] etc.), organic (e.g., viologens [67,68], organic redox dyes [69], polyaniline (PANI) [70], poly(3,4-ethylenedioxythiophene) (PEDOT) [71,72]), and hybrid (inorganic–inorganic, organic–organic, inorganic–organic) [73], composite/nanocomposite [74,75], resonant–cavity structures [76] (e.g., plasmonic [77] and Fabry–Perot [78] and 2D materials [79]), among others.

The ion transport layer is located between the electrochromic layer and the ion storage ones and has the function of transporting ions within the device. Conventional candidates for ion transport materials include lithium salt, ammonia salt, ionic liquid [80,81], polymers with ionic conductivity [82,83,84,85], and liquid crystals [80]. The ion storage layer is a complementary layer used to balance the charges by undergoing a reversible electrochemical redox process, corresponding to the reduction or oxidation of the electrochromic material in the electrochromic layer. The materials for the ion storage layer must have electrochemical reversibility, stability, capacity, and compatibility with the electrochromic layer [86,87,88]. NiO [89,90], CeO_2_ [91], IrO_2_ [92], TiO_2_ [34], VO_x_ [87,93], and radical polymers [88,94] are some examples of conventional ion storage materials. Currently, due to the collaborative efforts of several groups working in the field, research on electrochromic materials and devices has extended beyond colour changes in the visible range. Significant progress has been made in both theoretical understanding and the practical development of electrochromic materials and devices. Ongoing efforts aim to enhance the functionality and introduce innovative features to electrochromic devices, broadening their range of applications. For example, flexible electrochromic devices have been developed to meet the growing demand for flexible electronics, with applications in areas such as wearable devices and flexible displays. Additionally, there has been extensive research on electrochromic materials and devices designed to enable optical modulation in the near-infrared (NIR) and infrared (IR) regions. This research is vital for applications in energy-efficient smart windows, thermal regulation, and infrared camouflage [95,96,97].

In parallel, ongoing developments are focusing on systems that ensure effective electrolyte circulation or pumping, which is crucial for the stable operation of large-area smart windows using liquid electrolytes [98,99].

**Figure 1:**
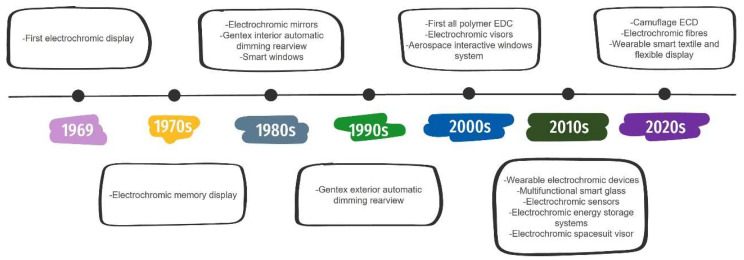
The evolution of electrochromic devices from single functionality to flexible and integrated systems. First electrochromic display [100] in 1969; electrochromic memory display [101] in the 1970s; electrochromic mirrors [102], Gentex interior automatic dimming rearview [103] and Smart windows [104,105] in the 1980s; Gentex exterior automatic dimming rearview mirror [103] in the 1990s; first, all polymer ECD [106], Electrochromic visors [107], Aerospace Interactive Window Systems [108] in the 2000s; wearable electrochromic devices [35], Multifunctional smart glass [109], Electrochromic sensors [20,21,110], Electrochromic energy storage systems [111], Electrochromic space suit visor [112] in the 2010s; camouflage ECD [25,36], Electrochromic fibres [37,113], wearable smart textile and flexible display [114] in the 2020s.

Figure 1 illustrates the progression of electrochromic devices, evolving from single-function systems to advanced multifunctional flexible devices.

In this review, the most relevant state of the art and applications of multifunctional electrochromic materials and devices are described, with a focus on functional technologies based on their properties. Following a consistent exploration of the basic features, properties, characterization, and figure of merit of electrochromic systems, a detailed description of the most significant applications in functional technologies is presented, enhancing the most recent ones, which could open a new framework for electrochromic effect applications. Finally, new perspectives for the future are discussed.

## 2. Materials and Methods

### 2.1. Classification of Electrochromic Materials

Electrochromic materials can be classified in various ways based on different criteria.

(I) Redox mode (electron transfer): electrochromic materials are classified as cathodic, which gains electrons and typically exhibits colouration, or anodic, which loses electrons and becomes coloured [115,116]. Some transition metal oxides, such as molybdenum trioxide (MoO_3_) and niobium pentoxide (Nb_2_O_5_), exhibit cathodic electrochromic behaviour, while nickel oxide (NiO) demonstrates anodic electrochromic behaviour.
(II) Colour change: (a) materials that exhibit at least one coloured and one bleached state, such as MoO_3_; (b) materials that display two distinct colour states, for example, polythiophenes switching from red to blue; (c) multicoloured electrochromic materials, including or not, a bleached state, and this category typically comprises polymers and copolymers [117,118].
(III) Solubility of the redox states: (a) materials where both the reduced state and oxidized state are soluble, some examples are organic molecules and metal complexes; (b) materials in which only one redox state is soluble, such as in the reversible electrodeposition of metals; (c) materials in which all redox states are solid (insoluble). In this type of electrochromic materials fall tungsten oxide (WO_3_), polymeric viologens, conducting polymers, etc. [119].
(IV) Relationship between redox-active units and chromophores in a more recent classification: (a) direct redox mode and (b) indirect redox mode [120]. In electrochromic materials with direct redox mode, chromophores and redox-active units are the same entity, and the colour change is caused by the electrochemically driven redox process of such units. In electrochromic materials with indirect redox, chromophores and redox-active are different entities. In this case, the colour change in chromophores is induced by energy transfer resulting from the electrochemically driven redox process of the redox-active units [121,122].
(V) Based on chemical composition and structure, electrochromic materials can be classified as inorganic, organic, composite/nanocomposite and hybrid materials [123].

### 2.2. Performance Indexes of Electrochromic Materials and Devices

**Figure 2:**
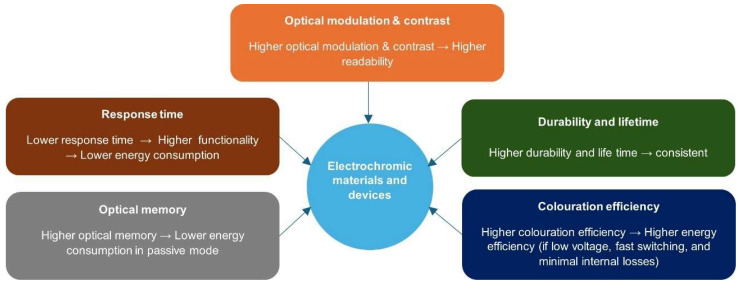
Typical performance indicators for electrochromic materials and related devices.

To facilitate the evaluation of electrochromic materials and devices, some indicators are usually used in literature to characterize their performance. As depicted in Figure 2, the performance indicators are optical modulation and contrast ratio, response time, optical memory, colouration efficiency and durability and lifetime, memory effect and response time.

#### 2.2.1. Optical Modulation (OM) and Contrast Ratio (CR)

Optical modulation and contrast ratio are important parameters to perform the colour-switching ability of an electrochromic material or device. Optical modulation is defined as the difference in transmittance *(*ΔT) or absorbance (ΔA) between the coloured and the bleached state at a specific wavelength (commonly at the wavelength of maximum colour modulation observed), Equation (1).

(1) $$OM=\Delta T=T_{bleached}-T_{coloured}\;or\;\Delta A=A_{coloured}-A_{bleached}$$

where $T_{bleached}$, $T_{coloured}$, $A_{coloured}$, and $A_{bleached}$ represent the transmittance (*T*), or absorbance (*A*) in the coloured state or bleached state. In the case of devices that work in reflectance mode, optical modulation is given by the difference in reflectivity.

The contrast ratio is comparable to the optical modulation (ΔT or ΔA). It is defined as the ratio of light intensity between the coloured state and the bleached state at a given wavelength Equation (2).

(2) $$CR=\frac{T_{bleached}}{T_{coloured}}\;or\;CR=\frac{A_{coloured}}{A_{bleached}}$$

For almost all electrochromic devices, high values of optic modulation and contrast ratio are desirable.

#### 2.2.2. Response Time

The response time, also known as switching time, is another important performance indicator in practical applications. It is defined as the required time for an electrochromic material or device to reach 90% of its full optical modulation from the bleached state to the coloured state (or vice versa) or between two colour states. Typically, a short response time is preferable. However, depending on the specific application, either a fast or slow response time may be acceptable. A response time on the order of minutes is acceptable for smart windows [124], while displays and mirrors, such as in Refs. [4,6,8,125], require switching times in the order of seconds or even milliseconds. The response time of an electrochromic device is highly influenced by the properties of its functional layers. For example, high ionic conductivity of the electrolytes enables fast ion transport between the electrochromic layer and the ion storage layer, thereby facilitating redox processes. On the other hand, ion diffusion within the active layers is governed by their structural characteristics, such as porosity, thickness and morphology. According to Marciel et al. [41], an increase in the thickness of the Mo–Nb–O active layer suggests longer colouration and bleaching times.

#### 2.2.3. Optical Memory Effect

The optical memory effect (also called the open-circuit memory effect) is the ability of an electrochromic material or device to maintain a coloured state after removing the electric field. Electrochromic materials or devices with strong memory effects are crucial for achieving zero-energy consumption electrochromic devices [126]. Significant efforts are being made to develop new ECDs with a sufficiently high open-circuit memory effect, ensuring that the coloured/bleached states can be maintained for several weeks or even months [2,126]. Some works in the literature report values ranging between 10 min and 180 days [126].

#### 2.2.4. Colouration Efficiency

Colouration efficiency (CE) correlates the change in optical density or optical modulation at the characteristic wavelength with the intercalated charges per active area, as defined in Equation (3).

(3) $$CE=\frac{\Delta OD}{\frac{Q}{S}}=\frac{log\left(\frac{T_{bleached}}{T_{coloured}}\right)}{\frac{Q}{S}}$$

where ΔOD is the change in optical density, Q is the injected charge, and S is the active area [6,124,127,128,129,130,131].

Colouration efficiency (CE) is a crucial indicator for assessing electrochromic materials and devices. Higher CE values indicate greater energy efficiency, which means that an ECD (electrochromic device) with a higher CE requires less charge to achieve the same level of optical modulation. Colouration efficiency (CE) is primarily influenced by the nature of the material type. The electrochromic process is based on electrochemical redox reactions and electron transfer between electrochromic materials and electrode surfaces. Thus, electrochromic reactions are governed by the insertion/extraction processes of electrons and ions, which are closely associated with the surface area and electrical properties of the active materials. For example, design-efficient and stable ion transport channels have been demonstrated to improve the performance, including the colouration efficiency of layered electrochromic materials [132]. Inorganic materials typically exhibit lower colouration efficiency, generally ranging from 10 to 100 cm^2^/C [133], whereas organic, hybrid, or composite materials tend to have higher colouration efficiencies, ranging from 100 to 1000 cm^2^/C [75,134,135]. However, a significant challenge affecting electrochromic performance reported in the literature is related to the unavailability of standardized evaluation procedures for comparing electrochromic materials and devices.

#### 2.2.5. Durability and Lifetime

Durability refers to the ability of electrochromic materials and devices to maintain their performance and functionality over time under extreme conditions. The American Society for Testing and Materials (ASTM) E2141 [136] and the International Organization for Standardization (ISO)ISO 18543 [137] have established standards for accelerated ageing tests of electrochromic devices. Critical factors influencing durability include temperature, humidity, photostability, oxygen exposure, and bendability.

Lifetime indicates the duration of an electrochromic material or device that can reliably function without experiencing significant degradation in its performance. It is measured in terms of the number of operational cycles or the total duration of effective use. The lifetime of an electrochromic device is mainly affected by the applied voltage, the type and properties of the electrolyte, work temperature, humidity, and external illumination (more prone to flexible devices due to the degradation of the substrate) [6]. In general, the lifetime of electrochromic devices depends on the sequence of a change in structural properties of electrochromic materials during electrochemical cycling, being important to control the electrons/ions transfer rates and insertion/extraction reversibility of the electrochromic films to extend the lifetime (measured for example as the retained optical modulation). For example, in electrochromic windows, it is expected (typically) to achieve at least 30,000 cycles [138], while displays aim for about a hundred cycles without significant optical degradation [7]. The durability and lifetime of electrochromic devices are closely linked to material properties, electrochemical mechanisms, and manufacturing processes.

### 2.3. Conventional and Emerging Electrochromic Materials

Electrochromic materials include inorganic, organic, composite/nanocomposite, and hybrid types. Table 1 summarizes the advantages and challenges of various conventional and emerging electrochromic materials [139,140,141,142,143,144].

Inorganic electrochromic materials involve non-carbon-containing compounds such as some transition metal oxides, mixed metal oxides, and Prussian blue. In general, inorganic electrochromic materials are characterized by good chemical and electrochemical stability in a wide range of working temperatures, high stability against UV radiation and good cyclability. Their drawbacks include slow switching times and limited colour tunability. Despite that, inorganic electrochromic materials are at the forefront of practical applications and broad commercialization [76]. Patel et al. [145] reported sputtered WO_3_ films with a maximum colouration efficiency of 96.96 cm^2^/C at 550 nm, 68.5% optical modulation in the visible region (at 550 nm) and 88.1% reversibility. These films demonstrated colouration and bleaching response times of 10 s and 24 s, respectively. The films showed good stability and durability over 1000 cycles across a temperature range of 5–50 °C.

Organic materials refer to carbon-containing compounds typically arranged in long carbon chains or rings such as viologens, conducting polymers, namely polyaniline (PANI) and poly(3,4-ethylenedioxythiophene (PEDOT). Organic electrochromic materials typically exhibit multiple bright colours, fast switching times, and superior colouration efficiency compared to inorganic materials. However, they also have drawbacks, including lower electrochemical stability [75,117], e.g., against UV radiation, diminished cyclability, and the need to use organic solvents during their processing, which pose flammability and toxicity risks [146]. Ding et al. [147] developed anion-doped PANI films for use in adaptive camouflage systems. These modified films showed excellent cycling stability, maintaining 78.49% of its initial optical modulation after 6000 cycles. Furthermore, they exhibit fast switching speeds, with bleaching and colouring times of 1.2 s and 1.5 s, respectively, measured at 660 nm.

Composites and nanocomposites are materials consisting of two or more components, where particles of one material are dispersed within the matrix of another. Usually, the components in a composite are not chemically bonded and have minimal interaction. Nanocomposites specifically refer to composites that contain dispersed particles at the nanoscale.

Recent studies have reported several promising advancements in nanocomposite materials. An et al. [148] demonstrated that the introduction of ITO nanoparticles significantly enhances the electrochromic properties of WO_3_ nanosheets. Su et al. [149] reported a PB/MnO_2_ composite material with a dual function showing remarkable areal capacitance (25.34 mF/cm^2^), outstanding electrochemical cycling stability (99.62% of the original surface capacitance after 1500 cycles) and a colouration efficiency of 2019.57 cm^2^/C at 480 nm.

Hybrid materials distinguish themselves from composites and nanocomposites by exhibiting a highly organized structure at the molecular and nanometric levels. The integration of different components leads to the formation of additional organized nanostructures. In a hybrid structure, the components interact and form chemical bonds, giving rise to nanosized structures with distinct characteristics. The properties of hybrid systems are influenced by the collective properties of their individual components, which may not combine in a straightforward additive manner. However, ideally, these interactions result in synergistic effects, leading to the emergence of new properties that are not observed in the original components. Hybrid materials can be composed of only organic or inorganic components. However, hybrid materials made up of chemically bonded organic and inorganic materials are of notable significance [123]. Recently, Park et al. [73] reported anodically colouring organic–inorganic hybrid electrochromic materials derived from phenothiazine cores. The hybrid film showed a response time of less than 1 s, a colour efficiency of 470 cm^2^/C at 630 nm, and long-term stability over 2000 cycles. In recent years, new hybrid materials have emerged, including metal–organic frameworks (MOFs). MOFs are highly ordered, three-dimensional crystalline organic–inorganic materials consisting of metal ions or clusters coordinated to organic ligands, which act as connecting nodes, forming a porous network with tunable pore sizes and chemical functionalities. MOFs are of great interest as electrochromic materials due to their high porosity, large surface area, and the ability to modify both metal nodes and organic ligands for tailored electrochromic properties [150,151]. Kumar et al. [152] synthesized isoreticular MOF thin films (Zn-PDI@FTO (PDI = perylene diimide)) exhibiting colouration efficiency of 941 cm^2^/C at 746 nm. These thin films retained more than 99% of electrochromic capacity after 100 reduction−oxidation cycles.

Extensive and ongoing research has been conducted to explore new materials and devices with improved electrochromic performance, leading to the discovery of promising new approaches. Optical resonators are good examples, and they have proven to be effective tools that provide strongly confined electromagnetic fields in ultra-small volumes, enhancing light-matter interactions. Several types of resonators based on distinct resonance mechanisms have been employed in the generation of structural colours, including plasmonic resonators, Mie resonators, Fabry-Pérot cavities, photonic crystal cavities and hybrid cavities [76,77]. Electrochromic plasmonic materials combine the properties of conventional electrochromic materials with the optical characteristics of plasmonic materials. They exhibit colour changes and light modulation via a synergistic combination of traditional electrochromic mechanisms (ion intercalation/deintercalation) and localized surface plasmon resonance (LSPR)-based light manipulation [76,77,153,154,155]. Zhang et al. [156] reported plasmonic oxygen-deficient TiO_2-x_ nanocrystals as effective single-component dual-band electrochromic material. These nanocrystals exhibit significant optical modulation at both visible and near-infrared (NIR) light transmittance, achieving remarkable optical modulation: 95.5% at visible (633 nm) and 90.5% at 1600 nm (NIR). Electrochromic switching speeds (colouration and bleaching times) were also reported at visible (630 nm) of 31.5 s and 9.6 s and at NIR (1600 nm) of 15.5 s and 3.4 s, respectively. The TiO_2-x_ nanocrystal electrode exhibits moderate colouration efficiency of 38.2 cm^2^/C at 633 nm and 112.7 cm^2^/C at 1600 nm. Furthermore, the TiO_2-x_ nanocrystal electrode demonstrates good long-term stability, retaining 95.6% of its capacity after 2000 cycles.

Reduced graphene oxide (rGO) is a kind of chemically derived graphene, also known as functionalized graphene, chemically modified graphene, and chemically converted graphene with good conductivity, high surface area and good mechanical strength [157]. The graphene reduction protocol has as its main objective the creation of graphene that is as close as possible to pristine graphene obtained from direct mechanical exfoliation of graphite layers. Despite the extensive research efforts, this goal remains far from being achievable. Residual functional groups and defects modify the structure of the carbon plane, making it inappropriate to label rGO, as the properties differ substantially [158,159]. Bhattacharjee et al. [160] reported the improved electrochromic properties of V_2_O_5_ incorporated within a rGO matrix using a wet chemical method, which resulted in the formation of V_2_O_5_ nanorods embedded in the rGO matrix. The optimized electrochromic film demonstrated switching times (colouring and bleaching times) of 6.2 s and 4.8 s, respectively, along with an optical modulation of approximately 54%, a coloured efficiency of about 347 cm^2^/C at 632 nm, and good electrochemical stability, withstanding up to 5000 cyclic voltammetry (CV) cycles with minimal degradation in the current response.

Covalent organics frameworks (COFs) consist of organic redox-active building blocks and electron-rich linkers. Due to the strong covalent bonds, COFs present rigid architectures with high crystallinity, resulting in good stability during electrochemical processes. The periodic distribution of pores and layered structure creates diffusion channels for ions, facilitating ion transport and, consequently, rapid switching times. Additionally, the quasi-aromatic feature of large-scale interlayer delocalized electrons enhances electron conductivity. In accordance with the linker design strategy, a donor–acceptor–donor electronic setup is possible to significantly improve intramolecular charge transfer and light absorption coefficients from the visible to near-infrared regions. These attributes of 2D COFs establish a robust foundation for good cycling stability as well as superior electrochemical and electrochromic performance. Despite significant advancements, COFs still face several challenges related to electronic delocalization, restricted pathways for electron transfer within the framework, and the interlayer structures connection (connected by van der Waals forces) that is vulnerable to random movement, affecting negatively the cycling stability [161,162]. Zhang et al. [163] reported three-dimensional metalated covalent organic frameworks (3D MCOFs) exhibiting notable electrochromic performance (cyclic stability of 93.6% retentions after 500 cycles, switching time less than 3 s and a colouration efficiency of 423 cm^2^/C at 700 nm. Coordination nanosheets (CONASHs) refer to a group of 2D polymers based on coordination compounds. CONASHs are compelling materials due to their diverse designs and functionalities achieved through numerous combinations of metal ions and organic ligands. They have a broad range of applications, including in the electrochromic field, that was first reported in 2015 [164,165]. Roy et al. [166] described the synthesisation of CONASH comprising three-arm terpyridine (3tpy)-based ligand and Fe (II) ions. The solid-state electrochromic device exhibited a colour change from pink to colourless state with an optical contrast of 53.1%, switching times (colouring and bleaching times) of 1.15 s and 2.49 s, respectively, long cycling stability with a loss of 4.98% in optical contrast after more than 1000 cycles, a colouration efficiency of 470.16 cm^2^/C at 556 nm, and memory effect of 50% retention of its colourless state during 25 min.

Transition metal carbides/nitrides/carbonitrides (MXenes) were first reported in 2011. Since then, they have garnered significant interest within the research community due to their unique properties, such as hydrophilicity and metal-like conductivity [167,168]. This emergent class of 2D materials are obtained from MAX phases with the general chemical formula M_n+1_AX_n_, where M represents a transition metal, A is usually any element from groups 12–16. X represents C, N, or, in some cases, the combination of both, and n is 1, 2 or 3^2^ [169]. MAX are precursors of MXenes. The first generation of MXenes was synthesized by selective etching of metal layers of MAX phases (layered transition metal carbides and carbonitrides) employing hydrofluoric acid as an etchant. Subsequently, several other synthesis approaches have been developed, such as selective etching using a mixture of fluorine salts and several acids, non-aqueous etchants, halogens, and molten salts. The general chemical formula of MXenes is M_n+1_X_n_ T_x_ (*n* = 1–4), where M is a transition metal. X represents C and/or N, and T_x_ are surface terminations of the outmost transition metal layers and are dependent on MXenes type and method of synthesis [79,170,171].

The electrochromic effect in MXenes was first demonstrated by Gogotsi in 2019 in a titanium carbide (Ti_3_C_2_T_x_) thin film [172]. Kumar et al. [173] presented the design of an all-organic multifunctional solid-state electrochromic supercapacitor using Ti_3_C_2_T_x_ doped with ethyl viologen and poly-3-hexylthiophene (P3HT) as a complementary electrode. The device showed an optical contrast of 85%, a colouration efficiency of 340 cm^2^/C at 515 nm, and switching times (coloured time and bleaching time) of 1 s. In addition, the device presented a specific moderated capacitance of 1.3 mF/cm^2^.

Table 2 summarizes the key performance parameters of electrochromic devices (discussed in Section 2.2), divided by the type of material, both conventional and advanced materials (Section 2.3), that were reported in the literature from 2020 to 2025. As mentioned above, each class of materials has advantages and challenges, and the electrochromic performance of each results from the combination of interrelated factors, such as their physical properties; electrochromic reaction mechanism used; material structure; process techniques and deposition methods; device design; quality of the device and sensitivity to environmental variables, such as humidity and temperature. Based on Table 2 and concerning colour changes, a multicolour ability is observed on hybrid, resonant cavity, and 2D materials (such as rGOs, COFs, CONASH, and MXenes). Inorganic materials exhibit more limited and monochromatic colour changes. Moreover, hybrid and resonant cavity systems present good optical modulation (~90%). Concerning the switching time, hybrid and MXenes electrochromic materials stand out for their ultrafast switching time (~1 s). Nanocomposite, hybrid, and 2D materials (such as rGOs, COFs, CONASH and MXenes) present higher colour efficiency. The maximum colour efficiency reported in this review is 2019.57 cm^2^/C at 480 nm for a composite material. In terms of durability and lifetime, inorganic materials appear to be the most promising within the temperature range of 0 °C to 50 °C.

### 2.4. Process Techniques and Deposition Methods for the Fabrication of Electrochromic Materials

**Figure 3:**
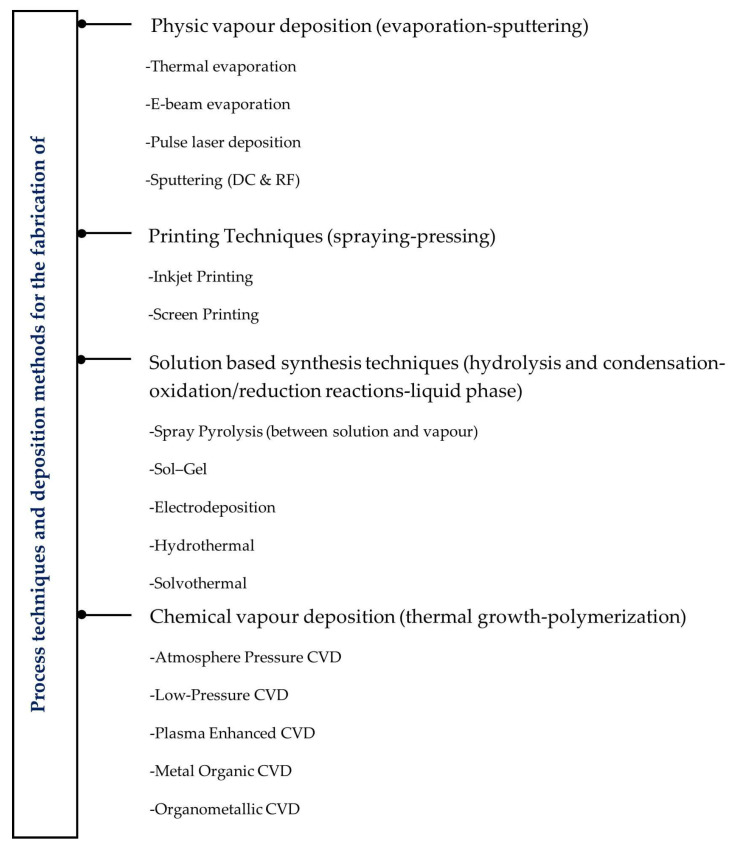
Schematic illustration of the classification of electrochromic films fabrication techniques.

Different techniques for the fabrication of electrochromic films can be employed, with the selection primarily influenced by several specific (and structure/application-related) factors. These include the desired film properties, substrate compatibility, deposition rate and thickness control, process complexity and equipment requirements, sustainability (eco-friendly processes), as well as other application-specific considerations such as cost-effectiveness, durability, and scalability. Figure 3 outlines key techniques available for the preparation of electrochromic films. These techniques are classified based on physical or chemical processes [176].

Physical processes include thermal evaporation [177,178], e-beam evaporation [179,180], pulse laser deposition [181,182], sputtering (DC and RF) [32,40], and printing techniques such as inkjet printing [183,184] and screen printing [185]. Chemical processes comprise gas-phase and solution-based syntheses techniques such as chemical vapour deposition [186], atomic layer deposition [187,188], spray pyrolysis [189], sol–gel [190,191], electrodeposition [192,193], solvothermal [194,195], and hydrothermal [196,197]. Each technique has its advantages and disadvantages. For example, physical deposition processes offer better characteristics compared to other techniques for most inorganic materials and some organic ones, providing films with high purity (no solvents or residual contaminants), as well as controlled thickness and uniformity. In addition, physical deposition methods are (usually) environmentally friendly. However, issues related to uniformity, especially for complex shapes (deep cavities and complex 3D shapes) can occur, as well as significant equipment and process costs must be considered [176,198]. Printed techniques are simple, low-cost, and scalable for large areas of production. However, these processes are traditionally not environmentally friendly and have limitations in printing fine patterns due to the printing line thickness and lateral resolution. A post-processing step such as thermal curing or sintering is needed, making this process relatively complex and expensive [183,199]. Chemical vapour deposition processes, in general, offer very high deposition rates, allowing the production of uniform, low-porosity thick films with high purity. Nevertheless, they require high temperatures and volatile precursors, which impose limitations on their application in flexible substrates [200]. Solution-based synthesis techniques, including spray pyrolysis and sol–gel, among others synthesis techniques, are scalable and versatile techniques. Spray pyrolysis is considered a cost-effective method operated in an open atmosphere process, facilitating adjustment and process visualization during deposition. However, spray pyrolysis is a complicated process with a low yield and oxidation risk under air atmosphere conditions, which can limit its efficiency and overall effectiveness in producing high-quality films [201]. The sol–gel method offers a straightforward, cost-effective, and efficient way to produce high-purity films. On the other hand, the prepared films exhibit weak stability, weak bonding, difficult porosity control, and risks of failure during the thermal process due to a mismatch of cracks from thermal expansion with the substrate [202]. Solvothermal and hydrothermal methods are simple chemical solution techniques performed in an autoclave for precise control over temperature and pressure, enabling the synthesis of high crystalline materials with controlled size and morphology. The hydrothermal method is considered cost-effective and environmentally friendly since water is used as a solvent. These techniques have certain drawbacks, particularly in the solvothermal method, which is more complex due to its reliance on typically organic solvents, often posing safety risks during the reaction process, along with the impossibility of studying in situ reactions due to their closed (or ‘black box’) system [203,204]. It is important to note that the selection of device substrate restricts certain process techniques and deposition methods due to temperature limitations and the associated risk of substrate degradation. Polymers and textiles typically exhibit degradation temperatures below 350 °C [205].

### 2.5. Conventional and Multifunctional Flexile Electrochromic Devices Architectures

Electrochromic devices, whether flexible or rigid ones, single-function or multifunctional, typically operate as electrochemical cells with two electrodes separated by an ion-conducting electrolyte. Different device architectures exist, and, in general, they are controlled by specialized power source electrochromic drivers, which modulate current or potential to induce electrochromic changes [116].

**Figure 4:**
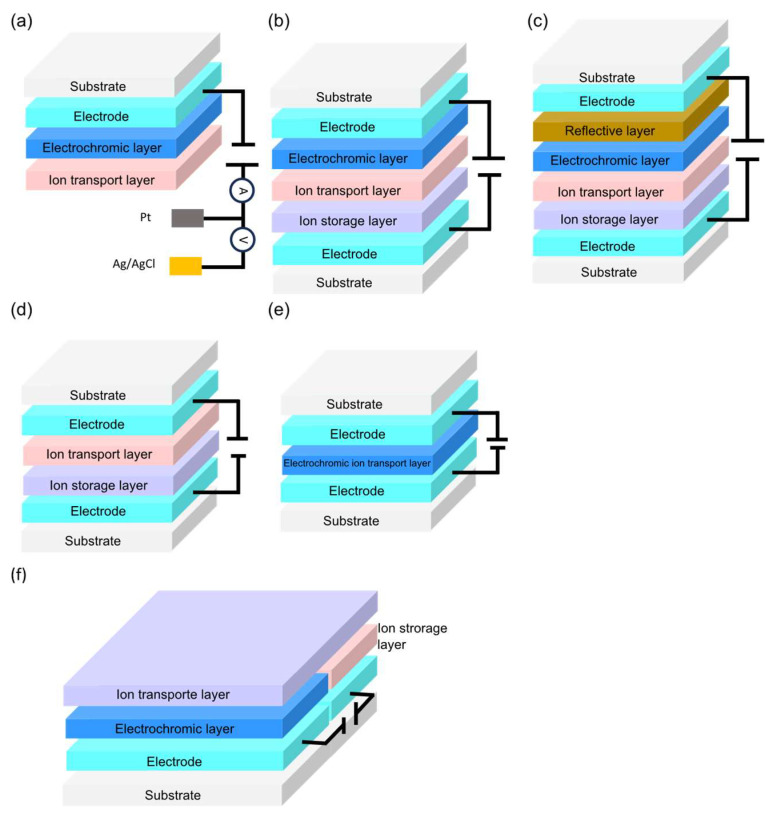
ECD structure. (**a**) Single-electrode structure. (**b**) Classic multilayer structure for ECD operating in transmittance/absorbance mode. (**c**) Classic multilayer structure for ECD operating in reflectance mode. (**d**) Reversible metal electrodeposition ECD (**e**) All-in-one structure. (**f**) Lateral multilayer ECD with five functional layers.

Conventional electrochromic devices are primarily constructed using different architectural structures such as single-electrode, multilayer, reversible metal electrodeposition, all-in-one, and laterally, as depicted in Figure 4a–f. The single-electrode structure (Figure 4a) is used in many current studies and applications, and it has been tested in an electrochemical cell set with a three-electrode configuration. The multilayer structure, Figure 4b, is based on a five-layer configuration and can be classified as an all-solid-state device [126,206,207,208], semisolid or gel-state device [209,210,211], and also as a liquid-state device [212,213].

Solid-state devices are of great interest for practical applications because they mitigate certain risks associated with semisolid and gel, and liquid-state devices, such as long-term sealing problems, safety issues related to the nature of the organic solvents used, bubble formations, inflammability, substantial decrease in ionic conductivity at zero temperature, and interfacial problems with the electrodes [206,214]. In this structure, the compact adherence of the electrochromic layer to the electrode layer allows for rapid electron transport and a short transport path. Applying a voltage (with a correspondent electrical current) to the EC devices causes the electrolyte species to diffuse towards the electrodes and insert into the electrochromic layer, resulting in visible colour and/or infrared emission changes. When the opposite voltage is applied, the ions are extracted from the EC layer, and the EC layer bleaches to the initial state [40]. ECDs can work in two modes of operation, either in absorbance, transmittance or reflectance, which can regulate the reflective spectra, as represented in Figure 4b,c. Smart windows and some display technologies work in transmission mode, where transparent conducting electrodes are used to regulate light absorption and transmission. Reflective ECD include displays, optical sensors, electrochromic mirrors, camouflage, and optoelectronic converters, and this configuration works with one transparent conducting electrode that remains clear while another is coated with a reflective layer to regulate the intensity of reflected light [125,215,216,217]. A reversible metal electrodeposition (RME) electrochromic configuration comprises an electrode, a counter electrode (which can be either an electrode or an ion storage layer), and a metal ion-based electrolyte in between, as illustrated in Figure 4d [192,193]. The all-in-one EC architecture (Figure 4e) comprises a symmetric device with an electrochromic material and a redox mediator dissolved in a liquid, solid, or semisolid electrolyte, resulting in a single layer electrochromic mixture that is sandwiched between two electrode substrates. This structure is restricted to electrochromic materials that are soluble in the electrolyte media (e.g., viologens) [210,214]. Applying a voltage to an all-in-one ECD causes ions in the electrochromic mixture layer to diffuse to the electrode surface or undergo a redox reaction with the redox mediator in the electrochromic mixture layer, resulting in colour changes [215]. On the other hand, in the lateral structure, the two electrodes and the EC layers are placed side by side on the same substrate rather than stacked. The ion conductor transport layer is placed on top of the EC layer and ion storage layer (Figure 4f). With the electrodes placed side by side, the risk of short circuits is reduced. However, electron transport occurs horizontally across the surface, extending the ionic diffusion path, resulting in reduced colour uniformity of the whole device [218].

## 3. Results

**Figure 5:**
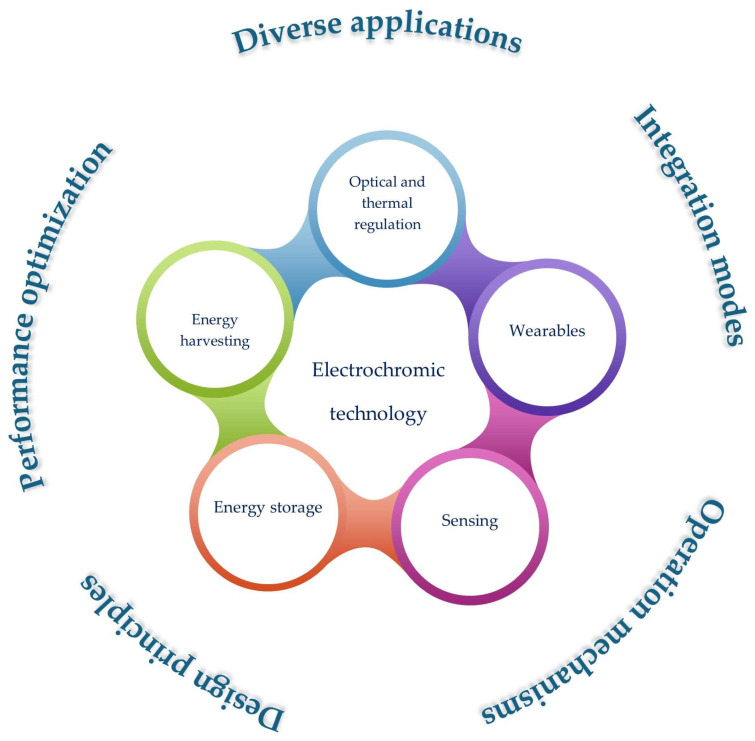
Schematic illustration of the integration of electrochromic technology with other advanced technologies.

The development of multifunctional flexible ECDs is a rapidly evolving and challenging area of research. These devices integrate a range of functions, including colour change, visible and infrared modulation, energy storage, energy harvesting, sensing capabilities, and display functions. However, ensuring flexibility and stretchability adds complexity to the design, as the materials and structures must maintain integrity and performance under bending and deformation. This unique combination of features makes the pursuit of such devices both thrilling and demanding, requiring innovative design strategies and advanced materials. The commercialization of conventional electrochromic devices, such as displays and smart windows, still faces several challenges, such as long switching times, poor cycle life, and limited colour options. However, significant advancements have been made in the past decade, thanks to the development of new EC materials at the nanoscale, the advance in fabrication techniques for electrodes, and improved device assembly protocols. In the domain of smart technologies, new possibilities have emerged for ECDs, enabling additional functions adjusted to various application scenarios. As a result of these performance enhancements and the expansion of application fields, multifunctional ECDs, including self-rechargeable electrochromic devices, electrochromic energy storage devices, deformable multicolour displays, and smart windows, among others, have been successfully demonstrated. Figure 5 illustrates the integration of electrochromic technology with other advanced technologies, along with the main strategies for improving multifunctional device performance. Electrochromic devices have progressed from single-function systems to multifunctional devices by integrating features such as energy harvesting (e.g., nanogenerators and solar cells) and energy storage (e.g., supercapacitors and electrochemical batteries). Through the selection of electrochromic materials capable of independently modulating the transmittance of visible and near-infrared light, it is possible to assemble electrochromic devices with thermal control functionality. Additionally, electrochromic sensors can be designed using materials that undergo voltage-driven, reversible optical changes in response to specific analytes or external stimuli. However, the integration of electrochromic technology with other advanced technologies poses several challenges, such as performance optimization, integration modes, operation mechanisms, and design principles. These aspects play a critical role in the development of multifunctional electrochromic devices (ECDs). In the following section, recent advances in multifunctional flexible ECDs are reviewed and discussed.

Table 3 summarizes recent multifunctional flexible electrochromic devices reported between 2020 and 2025, highlighting important device characteristics such as self-powering capability, energy storage ability, bistability, interactive colour/fluorescence changes, near-infrared modulation, and dynamic environmental adaptation. The table also details the electrochromic materials selected for device designs and the associated performance indicators, including driving voltage, colour change, optical modulation, switching time and cyclic stability.

### 3.1. Self-Powered Electrochromic Devices

The integration of electrochromic devices with energy storage systems is highly desirable across various applications, yet it presents significant challenges. Energy harvesting devices, such as piezoelectric nanogenerators (PENG), triboelectric nanogenerators (TENGs), and solar panels, among others, have been used to enable self-power of electrochromic devices [231,232,233,234].

PENG generates electrical energy through the piezoelectric effect, which is observed in specific materials when they are mechanically deformed by compression, tension or bending. In contrast, TENGs and solar panels are inherently dependent on environmental conditions. TENGs harvest mechanical energy from sources like wind, raindrops, human movement, water waves, sound, or magnetic-induced motion, while solar panels rely on sunlight. However, solar panels are ineffective during cloudy or rainy weather and cannot function at night, leading to limited performance and control.

A potential solution to overcome these limitations involves integrating energy storage components, such as batteries or supercapacitors, to ensure continuous operation.

#### 3.1.1. ECDs Powered by Nanogenerators

Nanogenerators are devices designed to capture small amounts of energy (mechanical, thermal, or other forms) generated, for example, by human motion. Nanogenerators can be integrated into clothing to harness energy from the movements of the hands, shoulders, feet, or arms, converting it into electrical energy at the nanoscale. This enables the powering of systems and devices such as self-power electrochromic wearables and self-power electrochromic sensors, among others [235,236]. Nanogenerators can be categorized based on their materials and operational principles into piezoelectric nanogenerators (PENGs) [219,237], triboelectric nanogenerators (TENGs) [238,239,240,241] and pyroelectric nanogenerators (Pyro-Ngs) [242,243].

**Figure 6:**
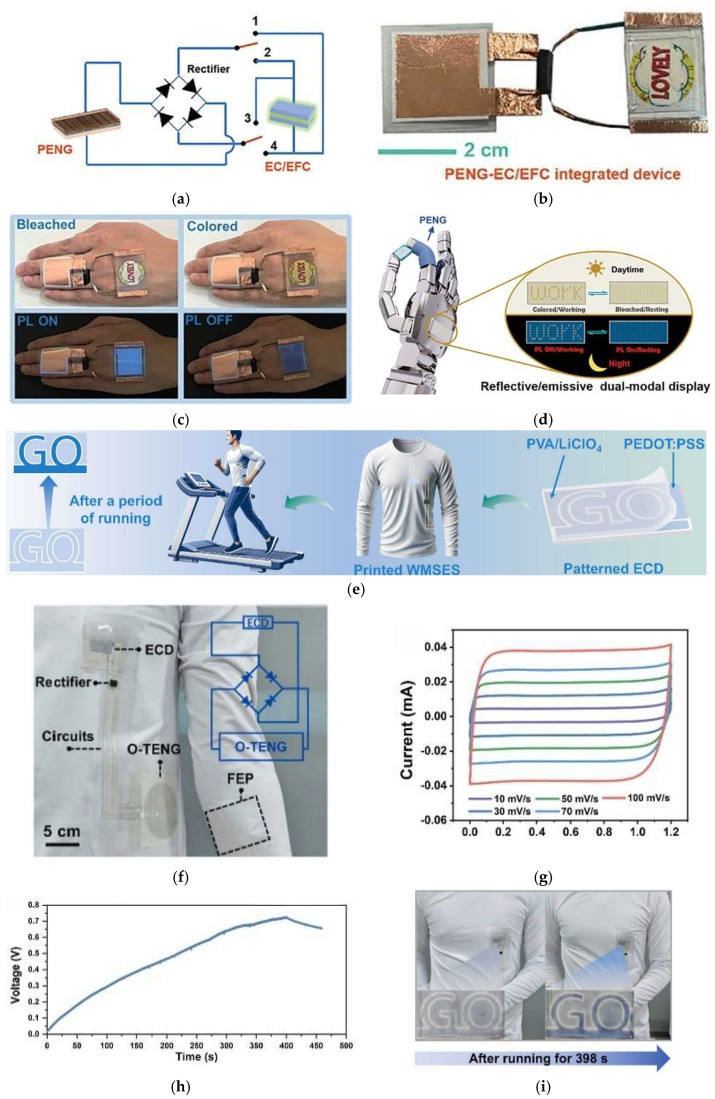
ECDs powered by Nanogenerators. (**a**) Circuit diagram of the integrated PENG and EC/EFC device linked with a rectifier [219]. (**b**,**c**) Photographs of the piezoelectric-driven EC/EFC device, along with images showing colour/fluorescence change at 452 nm in response to voltage fluctuations induced by continuous finger bending [219]. (**d**) Schematic illustration of safety precaution for a robotic hand based on the piezoelectric-driven EC/EFC integrated device [219]. (**e**) Schematic diagram of the patterned ECD, illustrating its structure, fabrication process, and integration into the WMSES [220]. (**f**) Visual image of the WMSES and circuit diagram [220]. (**g**) Cyclic voltammetry curves of the ECD at different scan rates [220]. (**h**) Self-charging curve of the WMSES voltage versus time [220]. (**i**) Visual images of the patterned ECD before and after running demonstrating colour change [220].

Zang et al. [219] presented a piezoelectric nanogenerator (PENG) driven electrochromic/electrofluorochromic (EC/EFC) integrated system, as illustrated in Figure 6a, capable of interactive colour and fluorescence modulation in response to human motion. This system uses an electroactive fluorescent ionic liquid based on triphenylamine and imidazole. Figure 6b,c shows photographs of the actual piezoelectric-driven EC/EFC device, including its operation in response to finger bending, demonstrating its potential for human motion sensing applications. Figure 6d shows a schematic of a potential application of the system as a safety indicator for robotic hands. The device exhibited remarkable electrochromic/electrofluorochromic performance, with low driving voltage, rapid switching speed (0.57–1.8 s), and good durability, retaining 91% of its performance after 10,000 cycles. Wang et al. [220] demonstrated the integration of a fully printed electrochromic device (ECD) with an omnidirectional triboelectric nanogenerator (O-TENG), incorporating printed micro-supercapacitors (MSCs) to form a wearable motion-interactive optical modulation system capable of autonomous operation. The system enables real-time visual feedback by converting motion (walking or running) into electrical energy, which is stored and subsequently used to trigger a reversible colour change in the ECD from light blue to dark blue, as illustrated in Figure 6e. The printed electrochromic cell is fabricated using screen-printing techniques and integrates functional components directly onto a flexible substrate. The device features a patterned electrochromic layer composed of PEDOT:PSS, which simultaneously serves as the conductive electrode and the active material responsible for the colour change. Specifically, the central “GO” label acts as the cathode, while the surrounding printed frame, also made of PEDOT:PSS, functions as the anode. A gel electrolyte composed of polyvinyl alcohol (PVA) and lithium perchlorate (LiClO_4_) is applied to cover both electrodes, completing the electrochromic system. The complete layout of the integrated system, including the O-TENG, printed circuit, and electrochromic module, is shown in Figure 6f, highlighting its compact, fabric-compatible configuration. Electrochemical characterization of the ECD is presented in Figure 6g, where the cyclic voltammetry curves exhibit nearly ideal rectangular shapes indicative of capacitive behaviour and efficient charge storage. The system’s energy harvesting and storage capability is confirmed in Figure 6h, which shows a self-charging curve where the voltage rises to 0.75 V after 398 s of arm motion. Finally, the effectiveness of motion-induced optical feedback is visually confirmed in Figure 6i, which displays photographs of the ECD before and after running, clearly demonstrating the transition in colour intensity and validating the self-powered, interactive nature of the device.

#### 3.1.2. ECDs Powered by Solar Energy

**Figure 7:**
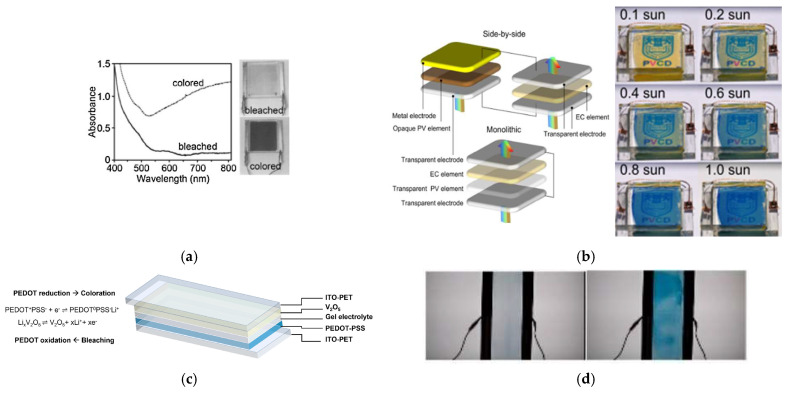
ECDs powered by solar energy (**a**) Absorption spectra and photographs of the first PECD under bleached and coloured states [251]. (**b**) Four-terminal side-by-side PV −EC device architecture: two terminals of PV element and two terminals of EC element connected via two external circles. Two terminal monolithic integrated PVCD architecture: only one external circle is required to connect top and bottom terminals [221]. (**c**) Schematic representation of the device, constituent layers and corresponding redox mechanism for colouration and bleaching [222]. (**d**) Visual images of clear and dark states of a 360 cm^2^ active area PEDOT-PSS/V_2_O_5_ assembled, under illumination conditions, cycled between +0.5 V and −4 V [222].

Flexible electrochromic devices (ECDs) powered by solar energy can convert sunlight into electrical energy, enabling the colour change in the ECDs. This combination of functionalities from both photovoltaic and electrochromic technologies gives rise to self-powered photoelectrochromic devices (PECDs) [244,245,246,247,248,249,250]. Presently, there are two main configurations of PECDs: the split-type (e.g., parallel side-by-side architecture), which combines two independent devices, and the tandem type (e.g., vertical tandem architecture) in a monolithic hierarchy configuration [221,251,252]. The split-type of PECDs is designed by integrating both the electrochromic and the photovoltaic device, while the tandem-type PECDs combine the photovoltaic electrode and the electrochromic layer in one device with several layers in one structure. The tandem-type PECDs architecture was first proposed by Bechinger et al. in 1960 [253]. This design features an independent dye-sensitized TiO_2_ photoanode paired with a WO_3_ electrochromic counter electrode. When the PECDs are illuminated under short-circuit conditions, the photoelectrons generated by the photoanode are injected into the WO_3_ film via the external circuit. The electron transfer is accompanied by the intercalation of Li^+^ ions into WO_3_, resulting in the formation of Li_x_WO_3_ and a corresponding colour change. Conversely, when the device is switched to open-circuit mode, and the light is blocked, the built-in photovoltage drives the extraction of electrons and Li^+^ ions from the Li_x_WO_3_ film via diffusion, leading to the bleaching of the PECD. Figure 7a presents the absorbance spectra, showing a progressive increase in optical density during colouration. Figure 7b provides visual confirmation through photographs showing the device transitioning from the bleached to the coloured state under illumination, demonstrating the reversible, light-driven optical modulation enabled by the PECD architecture. Cánovas-Saura et al. [222] developed a self-powered, flexible, all-printed electrochromic window with an active electrochromic area of 900 cm^2^. The device uses PEDOT:PSS as electrochromic material and a UV-curable, photo-crosslinkable gel as the electrolyte. A V_2_O_5_ layer serves as a transparent ion-storage counter electrode, and the system integrates organic solar modules as the integrated power supply. The overall device structure and working principle are illustrated in Figure 7c, which depicts the layer configuration and the autonomous energy transfer from the organic photovoltaic (OPV) module to the electrochromic multilayer structure. Figure 7d presents photographs of the device in its bleached and coloured states under illumination conditions while applying +0.5 V and −4 V.

### 3.2. Flexible Electrochromic Energy Storage Devices

Electrochromic energy storage devices, whether flexible or rigid, include electrochromic supercapacitors (ECSC) and electrochromic batteries (ECB). Among these, supercapacitors have been the most widely reported as flexible electrochromic energy storage devices. The fundamental difference between supercapacitors and batteries lies in their storage mechanisms, energy capacity, power constraints, charging speeds, and lifespan [254]. These devices (ECSC and ECB) are of particular interest for applications such as wearables, electrochromic windows, and electrochromic sunglasses because of their multifunctionality, including the ability to change colour in response to varying charge densities and their self-powering capabilities. It is important to note that the multifunctional integration of an electrochromic device with an energy storage device is feasible due to their shared characteristics, including similar material types, device configurations, and reaction mechanisms. Up to now, several electrochromic materials have been employed in the fabrication of ECSC and ECB as electrochromic materials. These include metal oxides, conducting polymers, inorganic–organic composites, and bimetallic materials or alloys [255].

#### 3.2.1. Flexible Electrochromic Supercapacitors

Electrochromic supercapacitors consist of an electrolyte that facilitates ion transport between the electrodes, a separator that acts as an electrical insulator, and two electrodes that typically exhibit electrochromic and supercapacitive properties, undergoing reversible colour changes when they charge or discharge. In some cases, the separator can also function as an electrolyte, combining the roles of ion transport and electrical insulation [236,256,257,258]. Electrochromic supercapacitors can be classified into electrochemical double-layer capacitors [259], pseudocapacitors [260], and hybrid supercapacitors [261] based on their energy storage mechanisms [262]. Their charge storage behaviour is governed by three fundamental mechanisms, which depend on the type of material used and synthesis method: (1) surface-controlled ion adsorption (excluding any Faradaic reactions); (2) charge storage via surface-controlled Faradaic reactions or ion intercalation; (3) a hybrid mechanism that combines both non-Faradaic and Faradaic charge storage processes [236,263].

**Figure 8:**
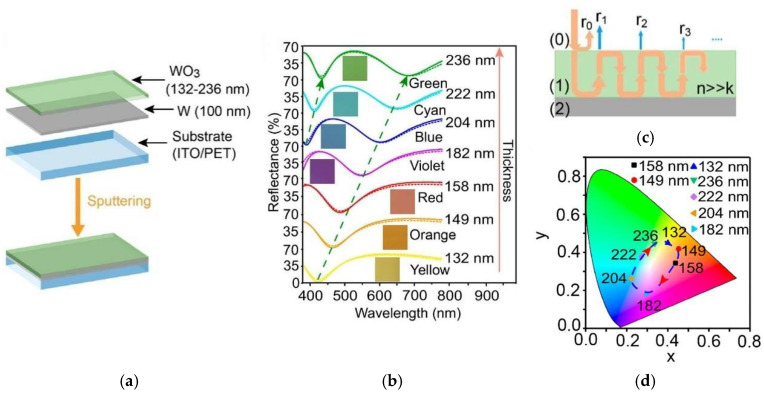
Preparation and Mechanism of F−P cavity-type electrochromic supercapacitor electrodes. (**a**) Schematic illustration of the layered structure of the device (**b**) Simulated (dashed line) and measured (solid line) reflection spectra. Inset: optical images of the F−P cavity-type electrochromic supercapacitor electrodes with different thicknesses of the WO_3_ layer. (**c**) Schematic representation of the light wave reflection processes of F−P cavity-type electrochromic supercapacitor electrodes. (**d**) CIE 1931 colour coordinates for the F−P cavity-type electrochromic supercapacitor electrodes with different WO_3_ layer thicknesses. Reprinted with permission from [78] Copyright (2020) American Chemical Society.

Chen et al. [78] introduced a novel approach for the aesthetic design of multifunctional electrochemical energy storage devices using Fabry–Perot (F-P) cavity-type electrochromic supercapacitors made from tungsten oxide. The layered structure of the F-P cavity-type electrochromic supercapacitor comprises an ITO-coated PET substrate layer, a tungsten (W) metallic layer and a tungsten oxide layer (WO_3_) with varied thickness. The metallic W metallic layer creates distinct interference resonances, resulting in multiple peaks and valleys in the spectral reflectance. The Fabry–Perot (F-P) cavity structure thus selectively absorbed certain wavelengths while reflecting others, enabling a wide range of vivid colours on the WO_3_ electrodes by varying the thickness of the WO_3_ layer. The device presented excellent electrochemical and electrochromic behaviour with a colouration efficiency of ~140 cm^2^/C, fast switching times, and a real capacitance of ~23.4 mF/cm^2^. Figure 8a–c illustrate the preparation and mechanism of F-P cavity-type electrochromic supercapacitor electrodes.

**Figure 9:**
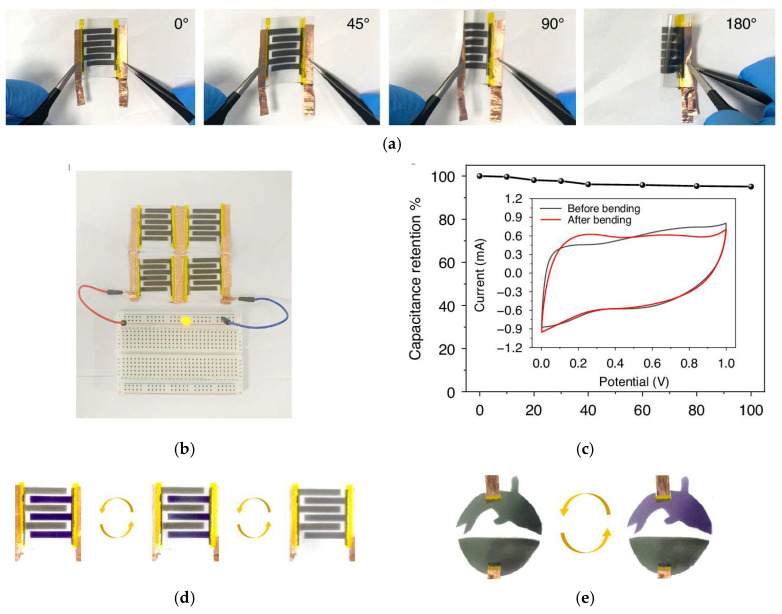
Electrochromic micro-supercapacitor. (**a**) Visual images of EMS at 0–180° bending angles. (**b**) Visual image of an LED illuminated by four EMS-3 devices in series and parallel (**c**) Capacitance retention of the EMS after 100 bending cycles at 180° (inset: CV curves before and after bending 100 times at a scan rate of 50 mVs^−1^. (**d**) EMS-3 under different charging states (fully charged, half charged, and empty from left to right). (**e**) On-surface patterned devices [223].

Guo et al. [223] developed a flexible electrochromic micro-supercapacitor utilizing Ti_3_C_2_ MXene, fabricated through a mask-assisted spray coating technique. By incorporating electrochromic ethyl viologen dibromide (EVB) into the electrolyte (1 M PVA/H_2_SO_4_ gel electrolyte), the device demonstrated a reversible colour change during the charging and discharging process. Combining the high electronic conductivity of MXene flakes and the rapid response kinetics of EVB, the device exhibited colouring/bleaching times of 2.6 s and 2.5 s, an optical contrast of 60%, a colouration efficiency of 209 cm^2^/C at 550 nm, and a real capacitance of 12.5 mF/cm^2^ with good mechanical stability (almost 100% capacitance retention after 100 bending cycles). Figure 9a illustrates the behaviour of the electrochromic micro-supercapacitor under different bending angles, showing its remarkable flexibility and mechanical stability. Figure 9b displays the EMS device powering light-emitting diodes (LEDs), clearly demonstrating its potential for powering electronic devices. Figure 9c presents the capacitance retention after 100 bending cycles at 180°, confirming the device’s durability under repeated mechanical stress, and Figure 9d highlights the device’s performance across three energy storage states (full, half-full, and empty), with real-time charge status indicated by colour intensity. Finally, Figure 9e shows various patterned device configurations, emphasizing their customizable design to suit different application needs.

#### 3.2.2. Flexible Electrochromic Batteries

Batteries and electrochromic devices share the same fundamental components: two electrodes and an electrolyte. In batteries, the charging and discharging process is governed by electrochemical redox reactions involving the insertion/extraction of ions and electrons. This phenomenon is similar to the reversible colour change observed in electrochromic devices under ion intercalation and deintercalation in response to an external electric stimulus [264].

The integration of these two technologies has led to the development of electrochromic energy storage devices, which combine electrochromic functionality with electrochemical energy storage functionalities by incorporating multifunctional materials [265,266]. Among the spectrum of battery technologies, secondary batteries (rechargeable), for example, lithium-ion batteries [267,268], have made outstanding progress. However, the limited availability of lithium and safety concerns related to organic systems present challenges to their ongoing development. In response, a range of alternative secondary batteries is emerging with rechargeable multivalent metal ions (e.g., Mg^2+^, Zn^2+^, Ca^2+^, Al^3+^) with great promise [265,269,270].

**Figure 10:**
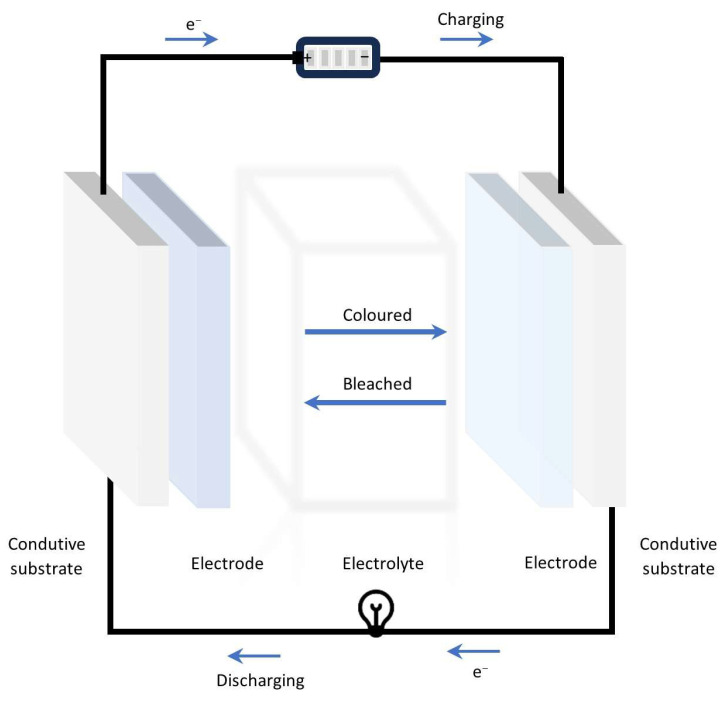
Schematic configuration of an electrochromic battery.

Figure 10 illustrates the schematic design of an electrochromic battery, which consists of conductive substrates that can be rigid or flexible (e.g., ITO-coated glass, FTO-coated glass, ITO-coated PTE), electrodes (anode and cathode), and an electrolyte. Conductive substrates provide structural support while ensuring efficient electron transport. The anode and cathode facilitate the electrochemical reactions necessary for energy storage and colour change. The electrolyte allows ion movement between the electrodes while maintaining electrical insulation. This configuration enables the battery to store and release energy while exhibiting a reversible colour change, providing real-time charge status visualization. The selection of appropriate electrochemically and electrochemically active materials.

Wu et al. [269] demonstrated a high-performance complementary electrochromic energy device based on the Wadsley–Roth Nb_18_W_16_O_93_ phase coupled with Prussian Blue using multivalent ions (Al^3+^/K^+^) in an aqueous electrolyte. The Nb_18_W_16_O_93_ and PB thin film electrodes were prepared on FTO glass. The device showed fast response (t_c_ = 1.8 s and t_b_ = 2.0 s), high colouration efficiency (~98.81 cm^2^/C at 632.8 nm) and good cycling stability (700 cycles with 94.68% retention). During the colouring/charging process, the ions are extracted from the Prussian Blue and inserted/embedded into the Nb_18_W_16_O_93_ electrode. When a reverse voltage is applied to the system, ions are extracted from the Nb_18_W_16_O_93_ electrode and intercalated into the PB electrode, and the system exhibits a bleached state. Galvanostatic charge/discharge curves of the Nb_18_W_16_O_93_ electrode displayed a maximum discharge specific capacity of 90.5 mAh/g at the current density of 0.05 mA/cm^2^.

Liu et al. [265] developed a Zn–ion electrochromic battery assembling a sodium vanadate (VO_Na+_) cathode, an ion-redistributing hydrogel enriched with anchored −SO_3_^−^ and −NH_3_^+^ (AMPHPL electrolyte), and a Zn anode. The VO_Na+_ cathode was coated on indium tin oxide-loaded polyethylene terephthalate (ITO-coated PET substrate). The Zn–ion electrochromic batteries showed reversible colour transition, shifting from orange (fully charged) to brown (partially charged and to green (discharged), allowing real-time energy monitoring. It delivers a high specific capacity of 302.4 mAh/g at 0.05 A/g and retains 96.3% of its capacity after 500 cycles at 3 A/g. Moreover, the Zn–ion electrochromic batteries remain operational (maintaining stable energy) under mechanical stress, including bending, rolling, knotting, and twisting, making them highly suitable for wearable electronics.

**Figure 11:**
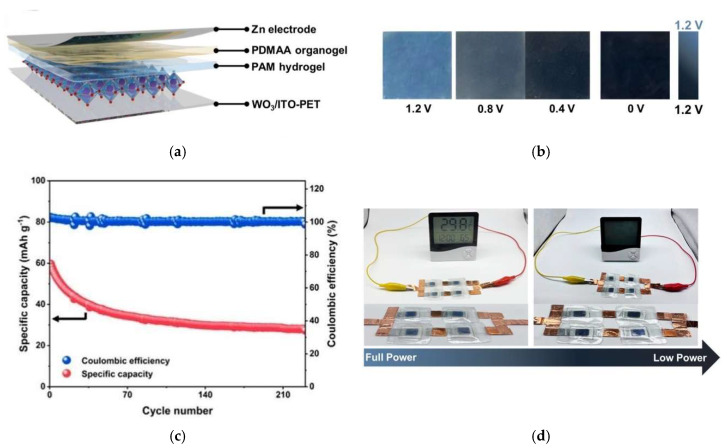
Rechargeable flexible electrochromic batterie. (**a**) Schematic illustration of the RZEBs configuration. (**b**) Optical images of the RZEBs under different applied voltages (from 1.2 V to 0 V). (**c**) Cycling stability of RZEBs employing a Janus gel electrolyte at a current density of 200 mA/g (**d**) Photographs showing the RZEBs powering an electronic thermo-hygrometer in both powered and unpowered states. Ref. [224] Copyright 2025 American Chemical Society.

Chen et al. [224] reported the fabrication of rechargeable electrochromic Zn–ion batteries (RZEBs), designed with a WO_3_ thin film as the electrochromic cathode deposited (ITO)-coated PET (Polyethylene Terephthalate) substrate, a Janus gel electrolyte (comprising a hydrophobic propylene carbonate-based poly(N,N-dimethylacrylamide) (PDMAA) organogel and a high ion conductivity polyacrylamide (PAM) hydrogel), and a zinc anode, as illustrated in Figure 11a. The electrochromic response of the device is depicted in Figure 11b), which presents optical images of the RZEBs under various applied voltages ranging from 1.2 V to 0 V. As the voltage decreases, the batterie exhibits a gradual colour transition, providing intuitive visual feedback on its state of charge. The device also demonstrated good cycling performance and electrochemical stability. Figure 11c displays the cycling performance of the RZEBs at a current density of 200 mA·g^−1^, demonstrating an average specific capacity of 33.5 mAh·g^−1^ and an average coulombic efficiency of 100% over 230 cycles. Furthermore, the practical functionality of the system is demonstrated in Figure 11d), where RZEBs are tested in powering an electronic thermo-hygrometer, with clear electrochromic contrast between charged and discharged states.

### 3.3. Flexible Multicolour Electrochromic Displays

**Figure 12:**
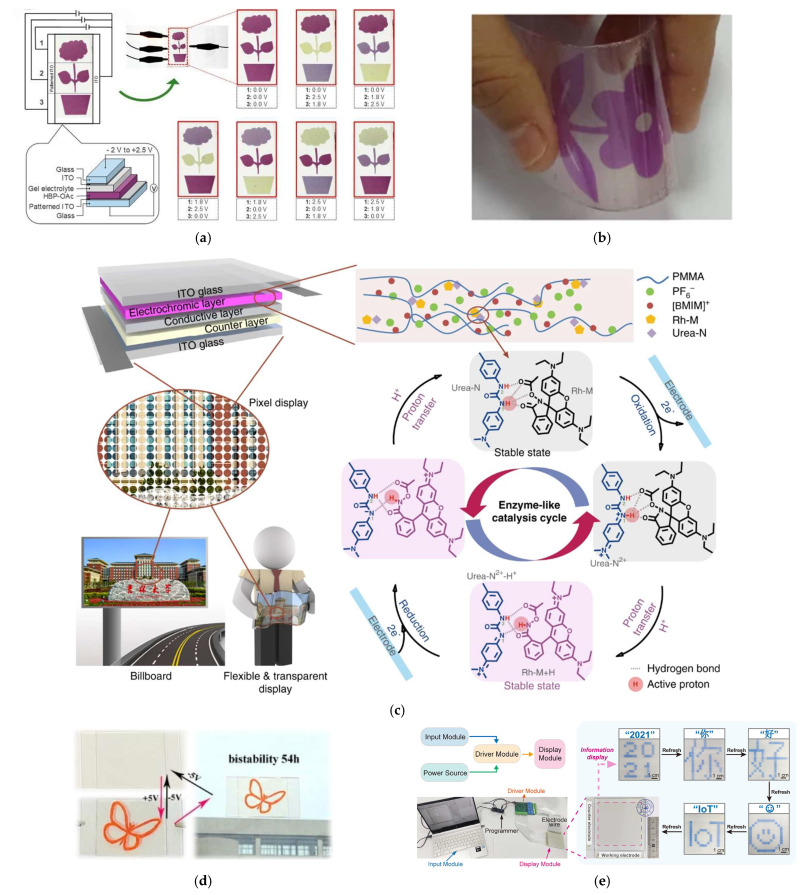
(**a**) Structure and images of an electrochromic segmented display presenting different colours at varying voltages. Adapted with permission from [225] Copyright 2020 American Chemical Society. (**b**) Flexible display prototype. Reproduced from Ref. [272]. (**c**) Schematic diagram and mechanism of a Urea-N+Rh-M energy-saving billboard and flexible display, illustrating the pixel display, the electrochromic device structure, the electrochromic layer composition, and the proposed mechanism. Reproduced from Ref. [272]. (**d**) Butterfly bistable energy-saving display window [226]. Copyright 2021 American Chemical Society. (**e**) Schematic and photography of a digitally controlled working module of transparent energy-efficient non-emissive pixelated display prototype demonstrating dynamic information refresh with numbers, Chinese characters, letters, and images [227].

Electrochromic displays, a type of non-emissive (passive) display, are gaining significant interest due to their low power consumption and excellent visibility, even in bright ambient light. They are categorized into two main types: segmented displays and pixel-based displays. Segmented displays are simple modes of display designed for presenting fixed graphics or alphanumeric characters [271]. The display content can be dynamically adjusted by controlling the electrical input. Bera et al. [225] demonstrated the fabrication of a multicolour electrochromic segmented display with different colour information at different voltages. The multicolour electrochromic display was designed by depositing a film of heterobimetallic supramolecular polymer having OAc^−^ as counteranion (HPB-OAc) onto the ITO substrate and using a lithium-based gel as the electrolyte, as illustrated in Figure 12a. The HPB-OAc film deposited onto the ITO substrate exhibited a maximum optical contrast of 52% at 575 nm, with response times of 0.72 s and 0.90 s for colouration and bleaching, respectively. It also demonstrated a colouration efficiency of 251 cm^2^/C at 575 nm and maintained stability over 10,000 electrochromic switching cycles. Zhang et al. [272] reported bistable flexible electrochromic segmented displays (Figure 12b) and pixel displays (Figure 12c) based on ITO electrodes, Urea-N+Rh-M electrochromic materials and [BMIM]PF6) electrolytes embedded on (poly(methylmethacrylate) (PMMA) incorporating Li-Nafion membranes for solid devices. The solid-state devices exhibit low switch-on voltage (+0.8 V) and distinctive colour gradients varying with voltages or time. They demonstrate a high colour efficiency of 430 cm^2^/C at 560 nm and rapid switching speeds, with colouring and bleaching times of 2.0 ms and 1.7 ms, respectively. Li et al. [226] demonstrated functional bistable electrochromic display windows, as shown in Figure 12d, using a highly elastic and bistable electrochromic ionic gel formed through a solution polymerization of a hydrogen-bonding cross-linking network. The ionic gel exhibited excellent tensile resilience, uniform colouring, reversible switching between coloured and bleached states under 24.3 s, a maximum transmittance change exceeding 80%, bistability of 54 h, stable performance over 500 cycles, and a colouration efficiency greater than 85.3 cm^2^/C at 501 nm.

Gu et al. [227] developed transparent, energy-efficient electrochromic displays, as shown in Figure 12e. The device was fabricated using interdigitated ITO finger as electrodes, photopatterned rhodamine-based non-nucleophilic electrochromic materials (RhNNEs) as an electrochromic layer and an electrolyte composed of PMMA, propylene carbonate (PC), TBAPF_6_, benzoquinone (BQ), and hydroquinone (HBQ) as electrolytes. To suppress optical signal crosstalk, a photolithographically defined pixel definition layer (PDL) was incorporated, confining the electrochromic materials and ion storage layers within individual pixel compartments. This design enabled precise pixel control, multicolour display capability and good optical modulation (~60% at 580 nm). The display exhibited low power consumption (~9.5 mW/cm^2^) and good bistability, retaining optical states for extended periods and maintaining stable performance over 20,000 switching cycles.

### 3.4. Flexible Smart Windows

**Figure 13:**
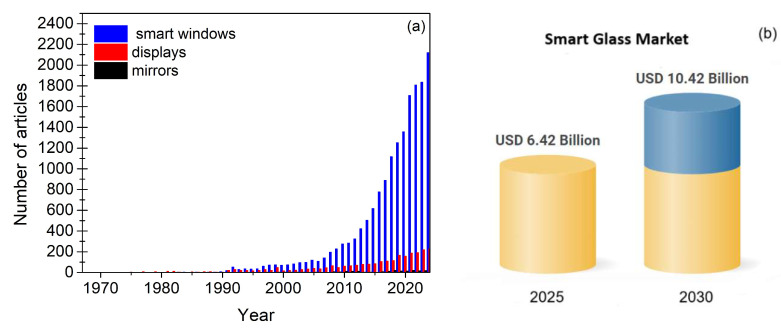
(**a**) Number of published articles on electrochromic devices from 1970 to 2024. Data was collected from the Web of Science. Topic (“electrochromic display” OR” electrochromic displays”/”electrochromic mirror” OR “electrochromic mirrors”/”smart window” OR “smart windows” OR “energy efficient windows”. (**b**) Global smart glass market (2025–2030): Market size in 2025 (yellow) and projected growth from 2025 to 2030 (blue) [278].

Reducing carbon dioxide and other greenhouse gas emissions has become a global imperative, driving the development of energy-efficient technologies across various sectors [273]. In developed countries, buildings are responsible for around 30–40% of total energy consumption, making them the largest energy consumers ahead of both industry and transportation. A significant portion of the energy is used by ventilation, heating, and air conditioning systems, with forecasts indicating a continued upward trend in demand over the next decades. Within this context, electrochromic devices, particularly smart windows, have emerged as a promising solution to reduce energy consumption and enhance indoor comfort [274,275,276,277]. As illustrated in Figure 13a,b, electrochromic windows continue to attract significant global research interest, while commercialization efforts are progressing with continuity and technical maturity.

**Figure 14:**
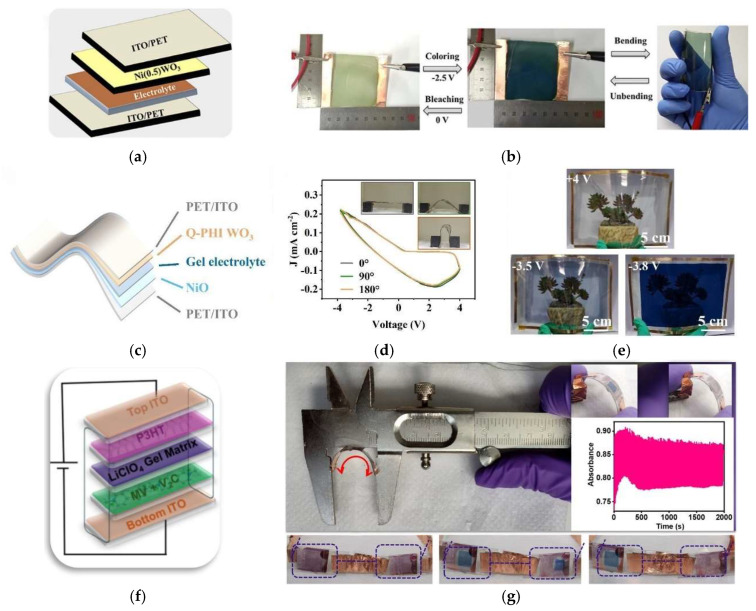
Flexible smart windows. (**a**) Schematic picture of the flexible electrochromic smart window using Ni(0.5)-WO_3_ [283]. (**b**) Ni(0.5)-WO_3_-based ECDs at beached and coloured state [283]. (**c**) Schematic representation of the structural configuration of the flexible Q-PHI WO_3_-based flexible electrochromic device [282]. (**d**) CV representative curves of the Q-PHI WO_3_-based flexible electrochromic device after bending at angles of 0°, 90°, and 180° [282]. (**e**) Digital photographs showing the colour states of the flexible Q-PHI/WO_3_-based electrochromic device under applied voltages of +4 V, –3.5 V, and –3.8 V [282]. (**f**) Schematic representation of the solid-state V_2_C ECD incorporating different electrochromic layers [228]. (**g**) Flexible V_2_C-based ECD demonstrating mechanical flexibility and different colouration/bleaching states in a flexible prototype [228].

Recent research suggests that multifunctional smart windows integrating energy storage systems offer even greater potential for reducing energy consumption [279,280]. However, traditional electrochromic smart windows mainly based on inorganic electrochromic materials (such as WO_3_ and V_2_O_5_, among other materials) continue to face challenges such as an excessive cost, structural complexity and poor colour tunability. In this context, the development of novel materials, including organic, composite, nanocomposite, hybrid, and other advanced electrochromic systems, is crucial to overcoming these challenges and further expanding the application of electrochromic smart windows. Flexible electrochromic smart windows represent an innovative advancement in the field, offering lightweight and the possibility of integration onto curved or irregular architectural surfaces [281,282]. Ahmad and Kim [283] designed a flexible electrochromic smart window (5 × 5 cm^2^) using Ni(0.5)-WO_3_ thin films deposited on ITO-coated PET substrates via the sol–gel method, using an EL-72 gel as the electrolyte, as illustrated in Figure 14a,b. The Ni(0.5)WO_3_-based electrochromic devices achieved a high colouration efficiency of 60.62 cm^2^/C and an enhanced optical contrast of 78.31%, with response times of 9.7 s and 17.2 s for colouration and bleaching, respectively. They also demonstrated solid long-term cycling stability, retaining performance over 25,000 cycles, and exhibited reliable mechanical flexibility, enduring up to 1000 bending cycles without any loss of functionality. Li et al. [282] introduced a novel quasiplanar heterointerface (Q-PHI) between the electrode and the electrochromic layer to enhance the performance of inorganic WO_3_-based electrochromic devices. A large-area flexible electrochromic smart window of 20 cm × 15 cm was successfully fabricated. The complete lightweight and flexible device comprised the Q-PHI heterointerface created by pre-treating the ITO surface with high energy oxygen ions before the deposition of the WO_3_ electrochromic layer, an electrolyte gel and a layer of NiO as ion complementary electrochromic layer as shown in Figure 14c. The flexible device demonstrated a switching time of 20.2 s for colouration and 9.4 s for bleaching, optical contrast of 67.6% at 1000 nm, high colouration efficiency of 354.4 cm^2^/C at 1000 nm, and long-term cycling stability, retaining 65.8% of its initial capacitance after 1000 consecutive cycles. Figure 14d shows CV curves of the flexible Q-PHI WO_3_ electrochromic device measured after mechanical bending at 0°, 90°, and 180°, demonstrating electrochemical stability under deformation, and Figure 14e display photos of the Q-PHI WO_3_-based flexible electrochromic device under various driving voltages.

The development of new electrochromic materials, such as MXenes, is important due to their good chemical stability, good conductivity, and fast response times, making them strong candidates to improve or replace traditional electrochromic materials. Saumya et al. [228] developed an enhanced solid-state smart window by combining materials from distinct families to optimize electrochromic performance (Figure 14f). The device incorporates poly(3-hexylthiophene-2,5-diyl) (P3HT) and methyl viologen dichloride (MV) as electrochromic active electrodes. To further boost performance, multilayered 2D V_2_C MXene was introduced as a dopant into the n-type MV layer, while a LiClO_4_-based gel matrix served as the electrolyte. The V_2_C ECD exhibited a visible colour transition from magenta to blue under ±1.5 V, with a colour contrast of 38% at 520 nm and the ability to modulate NIR transmittance at 850 nm by up to 12%, enabling passive heat shielding functionality. A high switching speed of less than 0.5 s was also achieved. In addition, the device demonstrated a colouration efficiency exceeding 800 cm^2^/C in the visible range and maintained stable operation for over 2000 s during cyclic testing. To confirm its real-world applicability, a flexible version of the device was also fabricated, demonstrating not only good mechanical flexibility but also its functional robustness under bending conditions, as shown in Figure 14g.

Recently, ITO-free flexible electrochromic devices have attracted significant attention due to the inherent limitations of indium tin oxide, such as its brittleness, high cost, relatively low conductivity, and poor adhesion to flexible substrates such as PET. Zhang et al. [268] reported a flexible ITO-free smart window using Ag@Au core–shell nanowires as flexible transparent electrodes. The resulting all-in-one electrochromic device exhibits an optical contrast of 41% at 605 nm, a colouration efficiency of 106 cm^2^/C, and good cycling stability, with only about 20% degradation in optical contrast after 4000 cycles.

### 3.5. Other Types of Multifunctional and Flexible Electrochromic Devices

**Figure 15:**
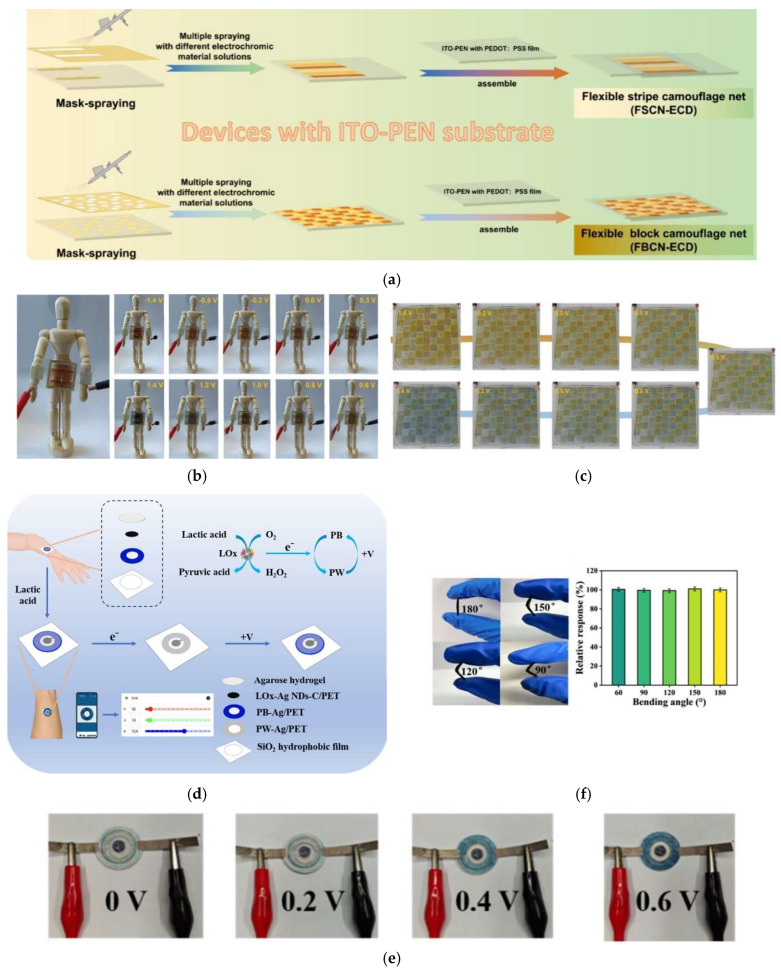
Multifunctional and flexible electrochromic devices. (**a**) Fabrication schematic of the flexible camouflage net device [229]. (**b**,**c**) Photograph of the FSCN-ECD and FBCN-ECD under different potentials (−1.4 to 1.4 V) [229]. (**c**) Photograph of the FBCB under different potentials [229]. (**d**) Schematic illustration of the wearable electrochromic sensor patch [230]. (**e**) Colouration restoration of the sensor patch under different applied voltages [230]. (**f**) Photographs of the sensor patch bent at different angles (90°, 120°, 150°, and 180°), along with the corresponding performance [230]. Copyright 2025 American Chemical Society.

In addition to the previously discussed examples, other types of flexible multifunctional electrochromic devices have been developed in recent years, particularly in the fields of sensing and adaptive camouflage systems. This advancement has significantly enhanced the applicability of flexible electrochromic devices, especially in advanced electronic and optoelectronic systems. However, electrochromic devices continue to face several challenges, including issues related to limited operating temperature range [284,285]. To respond to this problem, for example, Wu et al. [286] developed a dual-mode, temperature-dependent electrochromic device-based tungsten oxide capable of operating at an extremely low temperature. The incorporation of a PVA/EG-ZnCl_2_ organohydrogel electrolyte enabled stable electrochromic performance from −40 °C to 40 °C. The optimized device achieved transmittance modulation of 80.8% at 660 nm and retained 97.3% of its optical state after 32 h without power, demonstrating excellent low-temperature functionality and optical memory. Wang et al. [229] reported the fabrication of flexible camouflage net devices based on soluble yellow-to-green switching electrochromic materials, as illustrated in Figure 15a. The devices were assembled using ITO-coated polyethylene naphthalate (ITO-PEN) substrates, onto which different soluble electrochromic polymers (FTP, FEP, and FBP), synthesized from phenothiazine and ProDOT with various electron-donating units via direct (hetero)arylation polymerization were deposited. PEDOT was employed as the ion storage layer. Two flexible camouflage net designs were developed: a stripe-pattern and a block-pattern. Figure 15c,d shows the flexible stripe and block camouflage net electrochromic device (FSCN-ECD and FBCN-ECD) operating under different applied potentials ranging from −1.4 V to 1.4 V. Wei et al. [232] reports the development of a reusable, self-powered electrochromic sensor patch for on-site monitoring of lactic acid (LA) in human sweat, as illustrated in Figure 15d. The system combines enzymatic biofuel cells with electrochromic materials, enabling LA to act both as the target analyte and as a biofuel for power generation. The oxidation of LA by lactate oxidase generates electrons, which reduce Prussian Blue (PB) to Prussian White (PW), inducing a visible colour change, as shown in Figure 15e. The semiquantitative visual detection of LA can be performed through simple colour observation of the PB indicator. In contrast, quantitative detection is achieved by measuring the output current generated from electron transfer during the redox reaction. Therefore, on-site monitoring of LA can be achieved by capturing images with a mobile phone inside a lightbox and further using a colour recognition application to read RGB values. By increasing the applied voltage from 0 V to 0.6 V (Figure 15f), part of the Fe^2+^; in PW is oxidized to Fe^3+^, causing the restoration of the blue colour in the PB-Ag/PET electrode. The sensor exhibits a linear response to lactic acid concentrations ranging from 0.25 to 45 mmol/L in electrochemical measurements (limit of detection: 6.2 μmol/L), and from 1 to 45 mmol/L in optical measurements based on RGB analysis (limit of detection: 19.4 μmol/L). It demonstrates excellent selectivity against common interferents (e.g., glucose, uric acid), high repeatability (relative standard deviation (RSD) intra-assay results: 3.5% and inter-assay results of 3.8%), and long-term storage stability (signal retention >90% after 60 days at 4 °C). The patch sensor also maintains functional integrity after 50 reuse cycles and shows stable performance under mechanical bending, confirming its robustness and practical applicability.

## 4. Discussion

As pointed out, multifunctional flexible electrochromic devices (MFECDs) offer diverse functionality compared to traditional rigid glass-based ECDs, having the potential to be integrated into polymeric surfaces and in 3D technological applications. The latest advancements in MFECDs, such as self-powered smart windows that integrate energy storage and optical modulation in both visible and NIR regions, make it possible to regulate indoor lighting and temperature, thus reducing overall energy consumption; flexible and wearable electrochromic supercapacitors and electrochromic batteries with real-time energy monitoring; adaptative camouflage systems capable of dynamically altering their colour in response to environmental stimuli; flexible bistable electrochromic displays that can present dynamic information with ultra-low power consumption, and self-powered wearable electrochromic sensors that can detect signals without the need for an external power supply are detailed on Table 3. Ongoing developments are exploring the integration of electrochromic technology into everyday objects, such as smart wearables, touch-sensitive displays, and dynamic interactive user interfaces, taking advantage of its flexibility, tunable optical properties and self-powered capabilities. The most recent results demonstrate the adoption of ECDs in these areas, indicating their potential for broader applications in the future.

Nevertheless, as discussed, multifunctional flexible electrochromic devices are still at an early stage of development. Despite their excellent properties and potential applications, scientific developments are needed to solve key issues such as the scalability of manufacturing techniques, the long-term stability of multifunctional materials under real-world conditions, and their commercial viability (e.g., cost-effectiveness).

Among all the current problems, the scalability of fabrication techniques is probably the most significant challenge in the commercialization of multifunctional flexible electrochromic devices. This encompasses not only the capacity to achieve mass production of these devices at a low cost but also the need to keep multifunctional performance while relying on simple, efficient, and industry-compatible fabrication methods. Currently, the process methods and deposition methods for a multilayer electrochromic device include gas-phase methods, which afford precise control over film thickness and composition; solution-based methods, recognized for their simplicity and cost-effectiveness; physical deposition methods, which provide improved control but may pose a risk damaging polymeric substrates. Each of these techniques presents distinct advantages and challenges that impact the overall performance of electrochromic devices. Generally, these methods are expensive and remain confined to laboratory-scale applications.

There is an urgent need for advancements in large-scale preparation techniques that provide cost-effectiveness, the possibility of morphology manipulation, and precise management of materials, especially thickness and uniformity. Techniques of fabrication, such as integrating 3D printing and roll-to-roll [287,288], have emerged as promising approaches to achieve scalability. The method of 3D printing offers significant design freedom, enabling the fabrication of electrochromic devices with complex and customizable architecture. Meanwhile, roll-to-roll processing, due to its suitability for continuous, low-cost production, demonstrates strong potential for enabling the large-scale manufacturing of flexible electrochromic systems, addressing a critical requirement for their commercialization.

Nevertheless, scalability presents risks, such as the possibility of unpredictable and irreversible side reactions occurring due to the use of nanometric materials. Understanding these side reactions and electrochemical processes involved in EC materials and devices, particularly in the presence of multivalent ions, is of utmost importance, and it can be achieved through advanced in situ characterization techniques such as X-ray diffraction (XRD), Raman spectroscopy, and spectra-electrochemical synchronous investigation, among others.

On the other hand, future efforts to develop suitable materials with optimized conductivity, adhesion, chemical stability, and mechanical flexibility for 3D prints of ECDs and process improvements (roll-to-roll) to obtain better uniformity and adhesion between layers are crucial for commercialization. Materials such viologen/polyvinylalcohol(PVA)hydrogel inks [289], Prussian blue-viologen [290], poly(3,4 ethylenedioxythiophene):polystyrene sulfonate (PEDOT:PSS) [291] becomes of particular interest and belongs to the most recent examples of functional materials developed for 3D printing electrochromic materials with interesting results, opening new perspectives for more suitable devices. Moreover, multifunctional materials provide the advantage of simplifying device architecture by integrating multiple functions into a single material. This integration reduces the number of layers and interfaces, leading to lower production costs and improved compactness. However, using distinct materials for each function still offers advantages, mainly in terms of performance optimization.

Another important issue related to the long-term multifunctional materials’ stability under real-world conditions is that both the long-term stability and their additional functionalities, such as energy storage and thermal management, are crucial for the development of next-generation electrochromic devices. Based on this revision (e.g., Table 2 and Table 3), the synergies created using advanced materials (composite/nanocomposite, hybrid, and 2D materials electrochromic materials) significantly improve the device’s EC properties, adding versatility.

The optimization of multifunctional devices demands consideration of multiple critical factors.

The selection of high-performance materials (large optical modulation, fast switching speed, high colouration efficiency, and robust cycling stability), combined with the integration of composite materials, as discussed in Section 2.2, plays a crucial role in enhancing multifunctional performance. From a structural perspective, adopting multilayer architecture and incorporating multifunctional electrodes contribute to simplifying the overall device design, as mentioned before. In terms of electron/ion transport kinetics and redox reaction (e.g., electrochromic layer–electrolyte), interfaces greatly influence the electrochromic and electrochemical performance of an ECD being essential, optimize the stability of the interface and improve charge transfer efficiency by selecting electrolyte materials that ensure both functional and structural compatibility.

The development of solid-state electrolytes, more suitable for flexible electrochromic devices (ECDs), eliminates the risk of electrolyte leakage. These solid-state electrolytes must demonstrate adequate ionic conductivity at operating temperatures, excellent electrochemical stability, and appropriate deformability. However, the solid-state electrolytes reported to date exhibit low ionic conductivity and are prone to cracking under mechanical stress. Consequently, more efforts are needed to develop suitable solid-state- electrolytes to meet the requirements of stretchable/deformable ECDs. Another important challenge is related to the long-term cycling endurance and deformability of MFECDs. These devices need to respond under bending and stretching conditions, which increases the risk of premature performance degradation due to delamination/dissociation or leakage of the electrolyte. Li W et al. [292] propose thermal annealing to improve adhesion in rigid devices; however, organic polymer substrates cannot withstand those temperatures. This highlights the need to develop alternative strategies to ensure effective integration between different layers. Another important aspect related to the overall multifunctionality is the methodology used to measure ionic conductivity and the electrochemical stability (ESW) of electrolytes. It is essential to evaluate these parameters under conditions that closely mimic actual device applications. It is also highly recommended to study the potential profiles of the working electrode and counter electrodes during ECD operation (redox behaviour), as the performance of ECDs is evaluated in a two-electrode cell configuration (working and counter electrodes) by applying a constant current or voltage. Otherwise, the EC studies (cyclic voltammetry, chronoamperometry, etc.) are usually performed in a three-electrode configuration (working electrode, counter electrode and reference electrodes), and there is no information on the real scanned potential range of each working electrode in accordance with the cell voltage (E_cell_ = E_we_ − E_ce_) applied in the ECD (regardless the comparison to the reference electrode). The exploration of potential variation in each electrode of an ECD during the colouring/bleaching process can contribute to a better understanding of the EC mechanism and further develop high-performance multifunctional ECDs.

In the case of adopting distinct materials for each function, the development of flexible transparent electrodes with high conductivity, such as AZO, metal nanowires, carbon materials, metal grids, conducting polymers and MXene, open the doors for low-cost, lightweight and energy-efficient EC devices since it removes the cost associated with the production of rigid conductive ITO or FTO glass substrates. However, the transparent electrodes still need improvements related to electronic conductivity and transparency to compete with ITO/FTO glass substrates. Recent studies by Han Jisu et al. [293] proposed using oxide-metal oxide structures, combining indium tin oxide (ITO) and zinc-tin oxide (ZTO) with silver (Ag) for large-area flexible transparent conducting electrodes. This approach helps to minimize the brittleness associated with ITO/FTO, addressing the flexibility issues while maintaining the costs associated with the sputtering process.

Finally, the commercial viability (e.g., cost-effectiveness) of MFECDs holds both significant challenges and promising opportunities. The scalability of process techniques and deposition methods, operational stability under real-world conditions, and overall cost-efficiency are significant factors for successful commercialization. A major challenge lies in developing materials that combine high electrochromic performance with long-term durability, particularly under repeated cycling and exposure to harsh environmental conditions, without compromising multifunctional capabilities. To overcome these limitations, as discussed previously, current research focuses on optimizing materials and processes to increase stability and reduce costs. Advances in scalable production processes such as printing and roll-to-roll and solutions-based synthesis techniques are important advances for reducing the cost of ECD manufacturing, making them more realistic for commercialization. Regulations concerning CO_2_ emissions and market trends towards sustainable and smart technologies are favourable factors to the successful commercialization of MFECDs. Based on ongoing advancements, as demonstrated in this revision, it is expected that MFECDs will lead to a significant expansion of their practical applications soon, particularly in special niche applications where ECDs are unique. This progress will be driven by the optimization and integration of diverse electrochromic materials, the design of hybrid approaches, a deeper understanding of underlying mechanisms, and the development of innovative fabrication technologies.

## Figures and Tables

**Table 1:** Advantages and challenges of conventional and emerging electrochromic materials.

Electrochromic Materials		Advantages	Challenges
Conventional electrochromic materials	Inorganic Transition metal oxides, Prussian blue, etc.	-Long term stability -High optical contrast	-Slow switching time -Limited colour tunability
	Organic Viologens Conjugated polymers Metal–organic complex, etc.	-Rapid switching times -Multi and bright colours	-Insufficient long-term stability -Flammability and toxicity risks
Emerging advanced electrochromic materials	Composite/nanocomposite Dispersed nanoparticles on a matrix	Comprising the advantages/disadvantages of both organic and inorganic materials	
	Hybrid (organic–organic/inorganic–inorganic and organic–inorganic) Metal–organic frameworks (MOFs)		
	Optical resonators Plasmonic resonators Mie resonators Fabry–Perot cavities Photonic crystal cavities Hybrid cavities	-High optical contrast -Ultrafast switching times (milliseconds) -Multi colours	-Poor biostability -Inhomogeneous colours -Poor lifetime
	Emerging 2D materials Reduced graphene oxide (rGO)/ Coordination nanosheets (CONASHs)/ Covalent organics frameworks (COFs)/ Transition metal carbides/ Nitrides/carbonitrides (MXenes)	-Diversified structures -Customized functions -Multifunctionality	-Early-stage research

**Table 2:** Summary of the recent performance of electrochromic devices designed with different types of materials, as reported in the literature from 2020 to 2025.

	Structure EC Device EC Film	Applied Potential (V)	Colour Change	Optical Modulation	Response Time (s) t_c_/t_b_	Colouration Efficiency (cm^2^/C)	Durability and Lifetime (Cycles)	Working Temperature Range (°C)	Year|Ref.
Inorganic electrochromic devices	WO_3_/FTO/glass	−2.5 to 1	Transparent-Blue	68.5% at 550 nm	10 24	96.96 at 550 nm	1000 (100% electrochemical stability retained)	0 to 50	2025 [145]
	Nb-Mo-O 0.62-Mo/Nb/ITO/PET	−2 to 2	Light golden Dark golden	75% at 630 nm	15.3 7.5	10.30 at 630 nm	>15 (100% electrochemical stability retained)	Room temperature	2025 [41]
Organic electrochromic devices	pTSA-PANI/ITO/glass	−0.8 to 1.2	Yellow-Green	73.9% at 660 nm	1.5 1.2	66.65 at 660 nm	6000 (78.49% electrochemical stability retained)	-	2025 [147]
	EC5-H_2_Q/BP-poly(viologen)/ITO/glass	−1.4	Green	79% at 617 nm	46 11.5	67.2 at 617 nm	500 (92.6% initial contrast retained)	-	2022 [174]
		−1.9	Purple	64% at 534 nm	15 27	62.1 at 534 nm	500 (43.6% initial contrast retained)		
Composite /Nanocomposite electrochromic devices	Y24-ITO-WO_3_ nanosheets/ITO/glass	−0.7 to 0.7	Transparent-Blue	77.69% at 633 nm	16.7 12.9	196.5 at 633 nm	180	-	2023 [148]
	EESD1-PB/MnO_2_/ITO/glass	−1 to 1.8	Green-Blue	32% at 480	2.98 3.62	2019.57 at 480 nm	1500 (99.62% electrochemical stability retained)	-	2023 [149]
Hybrids electrochromic devices	MeO-2EPT/ATO/FTO/glass	0 to +1.5	Transparent-Greenish blue	24.81% at 630 nm	<1	470 at 630 nm	2000	-	2024 [73]
	Zn-XDI-MOFs/FTO/glass	−1.9 to −0.6 −1.4 to −0.7 −0.9 to −04	Multicolour	96.4% at 746 nm	1.6 2.6	941 at 746 nm	150 (98% electrochemical stability retained)	-	2023 [152]
Resonant cavity electrochroMic devices	TiO_2-x_ NCs/ITO/glass	3.5 to 1.5	Blue (bright/cool/dark)	95.5% at 633 nm	35.1 9.6	38.2 at 633 nm	2000 (95.6% capability retention)	-	2020 [156]
				77.5 at 1600 nm	15.5 3.4	112.7 at 1600 nm			
	ITO/Cu/ITO/CeO_2_/LiNbO_3_/WO_3_/Al/ITO/ Glass	−4 to 4	Multilcolour	-	2.6 2.8	64.02 at 590 nm	7200 (84% capability retention)	-	2024 [175]
2D materials electrochromic devices	V_2_O_5_ in rGO/ITO/glass	−1 to 1	Yellow-Green	54% at 632 nm	6.2 4.8	347 at 632 nm	5000	-	2024 [160]
	3D Ti-DHTA-P_y_M COFs/FTO/Glass	−0.33- to 0.33	Orange red-Olive green	38% at 700 nm	2.5 0.5	423 at 700 nm	500 (93.6% electrochemical stability retained)	-	2024 [163]
	3tpy−Fe CONASH/ITO glass	3 to −2	Pink–Colourless	53.2% at 556 nm	1.49 2.49	470.16 at 556 nm	1000 (90.7% electrochemical stability retained)	-	2020 [166]
	Ti_3_C_2_T_x_-MXene/ITO/glass	0.2 to −1.8	Magenta-Blue	13.5% at 515 nm	~1	340 at 515	100 (100% electrochemical stability retained)	-	2024 [173]

**Table 3:** Summary of some recent multifunctional flexible electrochromic devices reported in literature from 2020 to 2025.

Device Type	Characteristics	Electrochromic Material/Material with Additional Function/ Multifunctional Material	Driving Voltage (V)	Colour Change	Optical Modulation	Switching Time (s) T_c_/T_b_	Durability and Life Time	Year|Ref.
Piezoelectric-driven electrochromic/electrofluorochromic dual-mode display device	Interactive colour/fluorescence change for human motion indication	EFIL-TPA—electroactive fluorescent ionic liquid based on triphenylamine (TPA) and imidazole PENG-based on PVDF/BaTiO_3_	0 1	Transparent-Blue	62% at 474 nm	T-0.58/0.70 (500 cycles) F-0.57/1.8 (500 cycles)	10,000 cycles (96% ΔT retained/91% fluorescence on/off ratio retention)	2023| [219]
All-in-one wearable self-powered system	Wearable Motion-interactive self-powered Arial capacitance 1.1 mF/cm^2^	PEDOT: PSS (poly(3,4-ethylenedioxythiophene): poly(styrenesulfonate) Tribolectric generator-WPU/BaTiO_3_	0 1.2	Light blue-Dark blue	-	6.27 9.09	6000 s (94.2% current retention)	2025| [220]
Photovoltachromic smart window	Self-powered	(HV(TF-SI)_2_) heptyl viologen bis(trifluoromethylsulfonyl) imide PV-component	~0.6	Transparent-Blue	~40% contrast ratio at 600 nm	200 300	10,000 cycles (40% initial contrast retained)	2021| [221]
Portable photovoltaic-self-powered flexible electrochromic windows	Portable Self-powered	PEDOT-PSS/V_2_O_5_ Organic solar modules	−4 0.5	Transparent-Blue	25% contrast ratio at 650 nm	<30	-	2021| [222]
Fabry–Perot cavity type electrochromic supercapacitors	Display of multicolour states Energy storage capacity Arial capacitance 22.6–68.4 mF/cm^2^	Tungsten oxide (WO_3_)	−0.5 0	Multicolour	-	Several seconds	3000 cycles (92% capacitance retained)	2020| [78]
Flexible and wearable electrochromic microsupercapacitor (EMS2)	Camouflage Anticounterfeiting Display Arial capacitance 12.5 mF/cm^2^	Ethyl viologen dibromide (EVB) -2D Ti_3_C_2_MXene	0 1	Colorless-Deep purple	-	2.6 2.5	100 cycles (100% capacitance retained)	2024| [223]
Electrochromic Zn–ion batteries	Energy storage Powering electronic devices with real-time energy monitoring Specific capacity 43.64 mAh/g	Tungsten oxide (WO_3_)	0 1.2	Sky blue-Black	-	-	160 cycles (60.84% capacitance retained)	2025| [224]
Tunable multicolour electrochromic devices	Tunable multicolour display	HBP-OAc (Fe(II)/Os(II) polymer	−2 2.5	Purple violet greenish yellow	47% ΔT at 575 nm	0.98 1.45	100 cycles (95% ΔT retained)	2020| [225]
Bistable energy-saving flexible displays	Bistablility electrochromic modulation (>54 h)	Poly(hydroxypropyl acrylate) (PHPA)-PMMA ionic gels	−5 5	Colourless-red	80% at 501 nm	24.3 at 501 nm	>500 cycles	2021| [226]
Transparent non-emissive electrochromic pixelated display	Augmented reality application Bistability (30 days) Energy consumption at 9.5 μW/cm^2^	Rhodamine (RhNNE)	−1 3	blue-Magenta-yellow-greenish black	32.6% ΔT at 580 nm	0.9 1.2	>20,000 cycles	2023| [227]
Flexible smart window/3D vision goggles	Switchable colour and NIR modulation	Methyl viologen dichloride (MV) -2D V_2_C MXene	−1.5 1.5	Magenta-blue	34% of colour contrast at 520 nm 12.4% ΔT at 850 nm	4.2 0.7 5.8 0.2	200 cycles (100% ΔT retained)	2025| [228]
Adaptative camouflage nets	Dynamic environmental adaptation through voltage-controlled colour-switching	PEDOT-PSS FEP electrochromic polymer	−1.4 1.4	Yellow-green	23.42% ΔT at 650 nm	1.15 2.09	1200 cycles (78% contrast retained)	2024| [229]
Reusable self-power electrochromic sensor patch for on-site visualization monitoring of lactic acid	Portable Flexible self-powered Biofuel cell power density of 5.2 μW/cm^2^ Detection range: 1 to 45 mmol/L (colour based) 0.25 to 45 mmol/L (current based)	Prussian blue (PB)	−0.2 0.6	Blue-blue fade	-	400	≥50 cycles	2024| [230]

## Data Availability

No new data were created or analyzed in this study. Data sharing is not applicable to this article.
